# Synthesis and *in Vitro* Evaluation of New Nitro-Substituted Thiazolyl Hydrazone Derivatives as Anticandidal and Anticancer Agents

**DOI:** 10.3390/molecules190914809

**Published:** 2014-09-17

**Authors:** Mehlika Dilek Altıntop, Ahmet Özdemir, Gülhan Turan-Zitouni, Sinem Ilgın, Özlem Atlı, Fatih Demirci, Zafer Asım Kaplancıklı

**Affiliations:** 1 Department of Pharmaceutical Chemistry, Faculty of Pharmacy, Anadolu University, 26470 Eskişehir, Turkey; E-Mails: mdaltintop@anadolu.edu.tr (M.D.A.); ahmeto@anadolu.edu.tr (A.Ö.); gturan@anadolu.edu.tr (G.T.-Z.); zakaplan@anadolu.edu.tr (Z.A.K.); 2 Graduate School of Health Sciences, Anadolu University, 26470 Eskişehir, Turkey; 3 Department of Pharmaceutical Toxicology, Faculty of Pharmacy, Anadolu University, 26470 Eskişehir, Turkey; E-Mails: silgin@anadolu.edu.tr (S.I.); oatli@anadolu.edu.tr (Ö.A.); 4 Department of Pharmacognosy, Faculty of Pharmacy, Anadolu University, 26470 Eskişehir, Turkey

**Keywords:** thiazole, hydrazone, furan, anticancer activity, anticandidal activity

## Abstract

Fourteen new thiazolyl hydrazone derivatives were synthesized and evaluated for their anticandidal activity using a broth microdilution assay. Among the synthesized compounds, 2-[2-((5-(4-chloro-2-nitrophenyl)furan-2-yl)methylene)hydrazinyl]-4-(4-fluorophenyl)thiazole and 2-[2-((5-(4-chloro-2-nitrophenyl)furan-2-yl)methylene) hydrazinyl]-4-(4-methoxyphenyl)thiazole were found to be the most effective antifungal compounds against *Candida utilis*, with a MIC value of 250 µg/mL, when compared with fluconazole (MIC = 2 µg/mL). Additionally, the synthesized compounds were evaluated for their *in vitro* cytotoxic effects on the MCF-7 and NIH/3T3 cell lines. As a result, 2-[2-((5-(4-chloro-2-nitrophenyl)furan-2-yl)methylene)hydrazinyl]-4-(4-chlorophenyl)thiazole was identified as the most promising anticancer compound against MCF-7 cancer cells due to its inhibitory effects (IC_50_ = 125 µg/mL) and relatively low toxicity towards the NIH/3T3 cell line (IC_50_ > 500 µg/mL).

## 1. Introduction

In the last decades, the aging as well as increasingly cancer-susceptible population has resulted in a corresponding increase in demand for new anticancer agents. Despite the improvement in the cancer treatment, cancer still remains a major health concern as the second leading cause of death throughout the world after cardiovascular diseases [1,2,3].

Eukaryotic pathogens such as fungi pose a particular therapeutic challenge since they share a close evolutionary relationship with their human hosts [4]. Generally, the treatment of fungal infections, particularly those caused by drug-resistant *Candida* species, is often complicated due to high toxicity, low tolerability, or narrow spectrum of activity [4,5,6,7,8].

In the last few decades, the increased incidence of life-threatening fungal infections has led to the search for new effective antifungal agents which can inhibit the growth of pathogens or eradicate them and have no or relatively low toxicity to host cells [4,5,6,7,8,9,10,11]. It is well known that thiazoles are present in many biologically active compounds, including natural products. Penicillins are important naturally occurring compounds carrying a reduced thiazole (thiazolidine) ring system [12,13]. Thiazole ring system is also found in pharmaceutical agents such as sulfathiazole (an antimicrobial drug), abafungin (an antifungal drug). The clinical efficacy of tiazofurin and its analogues, and bleomycins (BLMs) has also pointed out the importance of thiazole moiety for the treatment of cancer [14,15,16,17,18,19,20,21,22,23].

Hydrazides-hydrazones have also attracted a great deal of interest due to their increased importance in medicinal chemistry [24,25]. Isoniazid, a hydrazide derivative, is the frontline drug currently employed in the treatment of tuberculosis [26]. Hydrazone derivatives of isoniazid and other hydrazides have been reported to display significant antimicrobial activity [24,25,26,27,28,29,30,31]. Many substituted hydrazone derivatives have also been synthesized and evaluated for their antitumor activity, and some promising results were reported [32,33,34]. Besides, nitrofurans represent an important class of antimicrobial agents [35]. Nifuroxazide, a nitrofuran antibacterial agent bearing a hydrazone moiety, is widely used as an intestinal antiseptic [24].

In an effort to develop potent anticandidal and anticancer agents, herein we describe the synthesis of a new series of nitro-substituted thiazolyl hydrazone derivatives, focusing on their anticandidal effects and cytotoxicity against the MCF-7 and NIH/3T3 cell lines.

## 2. Results and Discussion

**Scheme 1:**
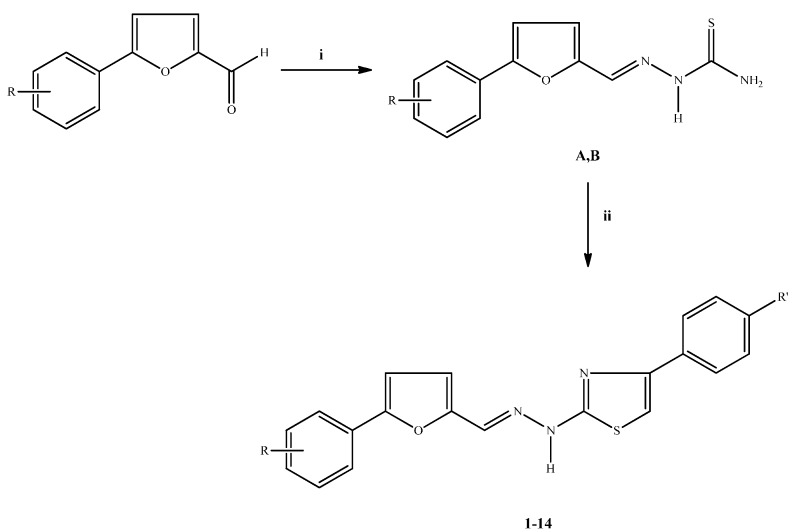
The synthetic route for the preparation of thiazolyl hydrazone derivatives **1**–**14**.

The synthesis of the thiazolyl hydrazone derivatives **1**–**14** was carried out according to the steps shown in Scheme 1. In the initial step, 5-arylfurfural thiosemicarbazones **A**/**B** were synthesized via the reaction of 5-arylfurfurals with thiosemicarbazide. The ring closure of the 5-arylfurfural thiosemicarbazones **A**/**B** with 2-bromoacetophenone derivatives afforded the new thiazolyl hydrazone derivatives **1**–**14**. The yields and melting points of the compounds are given in Table 1. IR, ^1^H-NMR, mass spectral data and elemental analyses were in agreement with the proposed structures of the compounds **1**–**14**.

*Reagents and conditions*: (i) NH_2_CSNHNH_2_, ethanol, reflux, 12 h; (ii) ArCOCH_2_Br, ethanol, reflux, 8 h.

**Table 1:** The yields and melting points (M.p.) of thiazolyl hydrazone derivatives **1**–**14**.

Compound	R	R'	Yield (%)	M.p. (°C)
**1**	*p*-NO_2_	H	80	213–214
**2**	*p*-NO_2_	NO_2_	85	252–254
**3**	*p*-NO_2_	F	82	241–242
**4**	*p*-NO_2_	Cl	81	229–231
**5**	*p*-NO_2_	Br	80	243–244
**6**	*p*-NO_2_	CH_3_	90	236–237
**7**	*p*-NO_2_	OCH_3_	90	232–233
**8**	*p*-Cl-*o*-NO_2_	H	75	194–195
**9**	*p*-Cl-*o*-NO_2_	NO_2_	95	216–220
**10**	*p*-Cl-*o*-NO_2_	F	90	208–209
**11**	*p*-Cl-*o*-NO_2_	Cl	88	186–187
**12**	*p*-Cl-*o*-NO_2_	Br	90	199–201
**13**	*p*-Cl-*o*-NO_2_	CH_3_	78	166–167
**14**	*p*-Cl-*o*-NO_2_	OCH_3_	90	206–207

The synthesized compounds were tested *in vitro* against pathogenic *Candida* species as shown in Table 2. Among these compounds, compounds **10** and **14** were found to be the most effective antifungal agents against *C. utilis*. Compounds **10** and **14** exhibited anticandidal activity with a MIC value of 250 µg/mL, whereas fluconazole exhibited antifungal activity with a MIC value of 2 µg/mL against *C. utilis.*

Additionally, all compounds **1**–**14** were evaluated for their cytotoxic effects on the MCF-7 human breast adenocarcinoma and NIH/3T3 mouse embryonic fibroblast cell lines as shown in Table 3 to determine their anticancer potential and selectivity. The most effective anticancer agent against the MCF-7 cell line was found to be compound **9** (IC_50_ = 102.58 µg/mL), followed by compounds **10** (IC_50_ = 121.79 µg/mL) and **11** (IC_50_ = 125 µg/mL) when compared with the reference cisplatin (IC_50_ = 31.2 µg/mL). IC_50_ values of compounds **9**, **10** and **11** against the NIH/3T3 cell line were 86.33 µg/mL, 250 µg/mL and >500 µg/mL, respectively. Due to its low toxicity towards the NIH/3T3 cell line (IC_50_ > 500 µg/mL), compound **11** can be identified as the most promising anticancer compound among the tested derivatives.

**Table 2:** The anticandidal activity of the compounds **1**–**14** expressed as MIC values (µg/mL).

Compound	*Candida* Species Tested					
	*C. albicans*	*C. utilis*	*C. tropicalis*	*C. krusei*	*C. parapsilosis*	*C. glabrata*
**1**	500	500	1000	500	500	1000
**2**	500	500	500	500	500	1000
**3**	500	500	500	500	500	1000
**4**	500	500	500	500	1000	500
**5**	500	500	500	1000	500	1000
**6**	500	500	1000	1000	500	500
**7**	500	500	500	500	500	1000
**8**	500	500	500	500	1000	1000
**9**	500	500	500	500	500	1000
**10**	500	250	500	1000	500	1000
**11**	500	500	500	1000	500	1000
**12**	500	1000	1000	1000	500	500
**13**	250	500	500	500	1000	500
**14**	250	250	250	500	500	500
**Fluconazole**	1	2	1	2	2	2

**Table 3:** The cytotoxic effects of the compounds **1**–**14** on the MCF-7 and NIH/3T3 cell lines.

Compound	IC_50_ (µg/mL)	
	MCF-7 Cell Line	NIH/3T3 Cell Line
**1**	>500	>500
**2**	>500	>500
**3**	500	250
**4**	>500	>500
**5**	>500	>500
**6**	>500	>500
**7**	>500	>500
**8**	>500	>500
**9**	102.58	86.33
**10**	121.79	250
**11**	125	>500
**12**	500	>500
**13**	500	>500
**14**	>500	>500
**Cisplatin**	31.2	330.58

On the other hand, compound **14** can be considered as the most promising anticandidal agent owing to its selective inhibitory effect on *C. utilis* and low toxicity towards NIH/3T3 cells. According to the cytotoxicity assay results, it can be concluded that functional groups at the 5th position of the furan ring and at the 4th position of the thiazole ring may have a crucial influence on cytotoxicity against MCF-7 and NIH/3T3 cell lines.

## 3. Experimental Section

### 3.1. General Information

All reagents were purchased from commercial suppliers and were used without further purification. Melting points were determined on an Electrothermal 9100 melting point apparatus (Weiss-Gallenkamp, Loughborough, UK) and are uncorrected. IR spectra were recorded on an IRPrestige-21 Fourier Transform Infrared spectrophotometer (Shimadzu, Tokyo, Japan). ^1^H-NMR spectra were recorded on a Bruker 400 MHz spectrometer (Bruker, Billerica, MA, USA) in DMSO-*d*_6_. Chemical shifts are expressed in parts per million (ppm) and tetramethylsilane was used as an internal standard. Mass spectra were recorded on a VG Quattro Mass spectrometer (Agilent, Apple Valley, MN, USA). Elemental analyses were performed on a Perkin Elmer EAL 240 elemental analyser (Perkin-Elmer, Norwalk, CT, USA). Starting materials and products were evaluated on TLC plates for their purity.

### 3.2. Chemistry: General Procedures for the Synthesis of Compounds **1**–**14**

#### 3.2.1. Synthesis of *5-(4-Nitrophenyl)furfural thiosemicarbazone* (**A**)/*5-(4-Chloro-2-nitrophenyl)furfural thiosemicarbazone* (**B**)

A mixture of 5-arylfurfural (0.025 mol) and thiosemicarbazide (0.025 mol) in ethanol (40 mL) was refluxed for 12 h. The reaction mixture was cooled and filtered [36].

#### 3.2.2. Synthesis of *2-[2-((5-(4-Nitrophenyl)furan-2-yl)methylene)hydrazinyl]-4-phenylthiazole/2-[2-((5-(4-chloro-2-nitrophenyl)furan-2-yl)methylene)hydrazinyl]-4-phenylthiazole* Derivatives **1**–**14**

A mixture of the appropriate 5-arylfurfural thiosemicarbazone (**A**/**B**) (0.001 mol) and 2-bromoacetophenone/4'-substituted-2-bromoacetophenone (0.001 mol) in ethanol (20 mL) was refluxed for 8 h. The reaction mixture was cooled and filtered.

*2-[2-((5-(4-Nitrophenyl)furan-2-yl)methylene)hydrazinyl]-4-phenylthiazole* (**1**): IR ν_max_ (cm^−1^): 3298.28 (N-H stretching), 3051.39 (aromatic C-H stretching), 2918.30, 2829.57 (C-H stretching), 1597.06, 1556.55, 1506.41 (C=N, C=C stretching and N-H bending), 1435.04, 1325.10 (C-H bending), 1280.73, 1213.23, 1107.14, 1016.49 (C-N, C-O stretching and aromatic C-H in plane bending), 910.40, 850.61, 798.53, 750.31, 721.38, 692.44 (aromatic C-H out of plane bending and C-S stretching). ^1^H-NMR δ (ppm): 7.03 (d, *J* = 3.6 Hz, 1H), 7.32–7.35 (m, 1H), 7.39 (s, 1H), 7.42–7.46 (m, 3H), 7.87–7.90 (m, 2H), 7.98–8.01 (m, 2H), 8.04 (s, 1H), 8.29–8.32 (m, 2H), 12.36 (brs, 1H). Anal. Calcd. for C_20_H_14_N_4_O_3_S: C, 61.53; H, 3.61; N, 14.35; Found: C, 61.52; H, 3.59; N, 14.36. MS (FAB): *m/z* [M+1]^+^ 391.

*4-(4-Nitrophenyl)-2-[2-((5-(4-nitrophenyl)furan-2-yl)methylene)hydrazinyl]thiazole* (**2**): IR ν_max_ (cm^−1^): 3292.49 (N-H stretching), 3116.97 (aromatic C-H stretching), 2987.74, 2900.94 (C-H stretching), 1598.99, 1575.84, 1514.12 (C=N, C=C stretching and N-H bending), 1415.75, 1344.38, 1327.03 (C-H bending), 1107.14, 1053.13 (C-N, C-O stretching and aromatic C-H in plane bending), 923.90, 850.61, 798.53, 750.31, 704.02 (aromatic C-H out of plane bending). ^1^H-NMR δ (ppm): 7.05 (d, *J* = 4.0 Hz, 1H), 7.46 (d, *J* = 4.0 Hz, 1H), 7.77 (s, 1H), 7.99–8.02 (m, 3H), 8.11–8.13 (m, 2H), 8.27–8.33 (m, 4H), 12.46 (brs, 1H). Anal. Calcd. for C_20_H_13_N_5_O_5_S: C, 55.17; H, 3.01; N, 16.08; Found: C, 55.15; H, 3.00; N, 16.08. MS (FAB): *m/z* [M+1]^+^ 436.

*4-(4-Fluorophenyl)-2-[2-((5-(4-nitrophenyl)furan-2-yl)methylene)hydrazinyl]thiazole* (**3**): IR ν_max_ (cm^−1^): 3120.82, 3053.32 (aromatic C-H stretching), 2920.23, 2835.36 (C-H stretching), 1597.06, 1556.55, 1510.26 (C=N, C=C stretching and N-H bending), 1328.95 (C-H bending), 1224.80, 1213.23, 1128.36, 1107.14, 1016.49 (C-N, C-O stretching and aromatic C-H in plane bending), 908.47, 835.18, 798.53, 740.67, 690.52 (aromatic C-H out of plane bending and C-S stretching). ^1^H-NMR δ (ppm): 7.00 (d, *J* = 3.6 Hz, 1H), 7.23–7.41 (m, 4H), 7.90–7.99 (m, 5H), 8.29 (d, *J* = 9.2 Hz, 2H), 12.37 (brs, 1H). Anal. Calcd. for C_20_H_13_FN_4_O_3_S: C, 58.82; H, 3.21; N, 13.72; Found: C, 58.80; H, 3.22; N, 13.71. MS (FAB): *m/z* [M+1]^+^ 409.

*4-(4-Chlorophenyl)-2-[2-((5-(4-nitrophenyl)furan-2-yl)methylene)hydrazinyl]thiazole* (**4**): IR ν_max_ (cm^−1^): 3315.63, 3188.33 (N-H stretching), 3118.90 (aromatic C-H stretching), 2972.31, 2870.08 (C-H stretching), 1587.42, 1568.13, 1504.48 (C=N, C=C stretching and N-H bending), 1438.90, 1323.17 (C-H bending), 1219.01, 1182.36, 1051.20, 1008.77 (C-N, C-O stretching and aromatic C-H in plane bending), 910.40, 848.68, 831.32, 783.10, 750.31, 688.59 (aromatic C-H out of plane bending and C-S stretching). ^1^H-NMR δ (ppm): 6.99 (d, *J* = 3.2 Hz, 1H), 7.39–7.48 (m, 4H), 7.88–7.98 (m, 5H), 8.28 (d, *J* = 8.4 Hz, 2H), 12.37 (brs, 1H)*.* Anal. Calcd. for C_20_H_13_ClN_4_O_3_S: C, 56.54; H, 3.08; N, 13.19; Found: C, 56.53; H, 3.09; N, 13.17. MS (FAB): *m/z* [M+1]^+^ 425.

*4-(4-Bromophenyl)-2-[2-((5-(4-nitrophenyl)furan-2-yl)methylene)hydrazinyl]thiazole* (**5**): IR ν_max_ (cm^−1^): 3186.40 (N-H stretching), 3116.97, 3062.96 (aromatic C-H stretching), 2972.31, 2864.29 (C-H stretching), 1585.49, 1571.99, 1504.48 (C=N, C=C stretching and N-H bending), 1438.90, 1323.17 (C-H bending), 1219.01, 1105.21, 1010.70 (C-N, C-O stretching and aromatic C-H in plane bending), 908.47, 846.75, 783.10, 748.38, 688.59 (aromatic C-H out of plane bending and C-S stretching). ^1^H-NMR δ (ppm): 6.98 (d, *J* = 4.0 Hz, 1H), 7.38–7.42 (m, 2H), 7.59–7.61 (m, 2H), 7.81–7.83 (m, 2H), 7.93–7.98 (m, 3H), 8.26–8.28 (m, 2H), 12.37 (brs, 1H). Anal. Calcd. for C_20_H_13_BrN_4_O_3_S: C, 51.18; H, 2.79; N, 11.94; Found: C, 51.17; H, 2.80; N, 11.93. MS (FAB): *m/z* [M+1]^+^ 470.

*2-[2-((5-(4-Nitrophenyl)furan-2-yl)methylene)hydrazinyl]-4-(p-tolyl)thiazole* (**6**): IR ν_max_ (cm^−1^): 3302.13, 3273.20 (N-H stretching), 3113.11 (aromatic C-H stretching), 2916.37 (C-H stretching), 1579.70, 1560.41, 1504.48 (C=N, C=C stretching and N-H bending), 1319.31 (C-H bending), 1213.23, 1138.00, 1105.21, 1024.20 (C-N, C-O stretching and aromatic C-H in plane bending), 850.61, 800.46, 729.09, 692.44 (aromatic C-H out of plane bending and C-S stretching). ^1^H-NMR δ (ppm): 2.33 (s, 3H), 7.00 (d, *J* = 3.6 Hz, 1H), 7.22–7.28 (m, 3H), 7.42 (d, *J* = 3.6 Hz, 1H), 7.76 (d, *J* = 8.4 Hz, 2H), 7.96–7.98 (m, 3H), 8.30 (d, *J* = 9.2 Hz, 2H), 12.34 (brs, 1H). Anal. Calcd. for C_21_H_16_N_4_O_3_S: C, 62.36; H, 3.99; N, 13.85; Found: C, 62.35; H, 3.98; N, 13.87. MS (FAB): *m/z* [M+1]^+^ 405.

*4-(4-Methoxyphenyl)-2-[2-((5-(4-nitrophenyl)furan-2-yl)methylene)hydrazinyl]thiazole* (**7**): IR ν_max_ (cm^−1^): 3120.82, 3072.60 (aromatic C-H stretching), 2933.73, 2821.86 (C-H stretching), 1618.28, 1597.06, 1506.41 (C=N, C=C stretching and N-H bending), 1330.88 (C-H bending), 1251.80, 1186.22, 1109.07, 1024.20 (C-N, C-O stretching and aromatic C-H in plane bending), 921.97, 850.61, 829.39, 796.60, 752.24 (aromatic C-H out of plane bending). ^1^H-NMR δ (ppm): 3.80 (s, 3H), 6.98–7.04 (m, 3H), 7.22 (s, 1H), 7.45 (d, *J* = 3.6 Hz, 1H), 7.80 (d, *J* = 9.2 Hz, 2H), 7.98–8.05 (m, 3H), 8.31 (d, *J* = 8.8 Hz, 2H), 12.35 (brs, 1H). Anal. Calcd. for C_21_H_16_N_4_O_4_S: C, 59.99; H, 3.84; N, 13.33; Found: C, 59.98; H, 3.84; N, 13.35. MS (FAB): *m/z* [M+1]^+^ 421.

*2-[2-((5-(4-Chloro-2-nitrophenyl)furan-2-yl)methylene)hydrazinyl]-4-phenylthiazole* (**8**): IR ν_max_ (cm^−1^): 3273.20 (N-H stretching), 3088.03 (aromatic C-H stretching), 2927.94, 2835.36 (C-H stretching), 1625.99, 1527.62, 1487.12, 1465.90 (C=N, C=C stretching and N-H bending), 1352.10 (C-H bending), 1249.87, 1099.43, 1026.13 (C-N, C-O stretching and aromatic C-H in plane bending), 983.70, 923.90, 879.54, 842.89, 798.53, 765.74, 748.38, 692.44 (aromatic C-H out of plane bending and C-S stretching). ^1^H-NMR δ (ppm): 6.99–7.07 (m, 2H), 7.12 (d, *J* = 3.6 Hz, 1H), 7.29–7.40 (m, 1H), 7.42–7.46 (m, 2H), 7.75–7.92 (m, 2H), 7.95 (d, *J* = 8.8 Hz, 1H), 7.99–8.06 (m, 2H), 8.13–8.15 (m, 1H), 12.37 (brs, 1H). Anal. Calcd. for C_20_H_13_ClN_4_O_3_S: C, 56.54; H, 3.08; N, 13.19; Found: C, 56.53; H, 3.09; N, 13.17. MS (FAB): *m/z* [M+1]^+^ 425.

*2-[2-((5-(4-Chloro-2-nitrophenyl)furan-2-yl)methylene)hydrazinyl]-4-(4-nitrophenyl)thiazole* (**9**): IR ν_max_ (cm^−1^): 3217.27 (N-H stretching), 3115.04, 3068.75 (aromatic C-H stretching), 1625.99, 1598.99, 1562.34, 1510.26, 1467.83 (C=N, C=C stretching and N-H bending), 1338.60 (C-H bending), 1257.59, 1211.30, 1136.07, 1111.00, 1028.06 (C-N, C-O stretching and aromatic C-H in plane bending), 893.04, 852.54, 781.17, 729.09 (aromatic C-H out of plane bending). ^1^H-NMR δ (ppm): 6.98 (d, *J* = 3.6 Hz, 1H), 7.11 (d, *J* = 3.6 Hz, 1H), 7.75 (s, 1H), 7.82–7.85 (m, 1H), 7.93–7.95 (m, 2H), 8.10–8.13 (m, 3H), 8.28 (d, *J* = 8.8 Hz, 2H), 12.46 (brs, 1H). Anal. Calcd. For C_20_H_12_ClN_5_O_5_S: C, 51.12; H, 2.57; N, 14.91; Found: C, 51.11; H, 2.56; N, 14.90. MS (FAB): *m/z* [M+1]^+^ 470.

*2-[2-((5-(4-Chloro-2-nitrophenyl)furan-2-yl)methylene)hydrazinyl]-4-(4-fluorophenyl)thiazole* (**10**): IR ν_max_ (cm^−1^): 3126.61, 3086.11 (aromatic C-H stretching), 2929.87, 2858.51 (C-H stretching), 1622.13, 1568.13, 1531.48, 1483.26 (C=N, C=C stretching and N-H bending), 1357.89 (C-H bending), 1236.37, 1219.01, 1153.43, 1114.86, 1035.77 (C-N, C-O stretching and aromatic C-H in plane bending), 881.47, 833.25, 752.24, 707.88 (aromatic C-H out of plane bending). ^1^H-NMR δ (ppm): 6.97 (d, *J* = 3.6 Hz, 1H), 7.11 (d, *J* = 3.6 Hz, 1H), 7.25 (t, *J*_1_ = 9.2 Hz, *J*_2_ = 8.8 Hz, 2H), 7.36 (s, 1H), 7.83–7.95 (m, 5H), 8.13–8.14 (m, 1H), 12.37 (brs, 1H). Anal. Calcd. for C_20_H_12_ClFN_4_O_3_S: C, 54.24; H, 2.73; N, 12.65; Found: C, 54.24; H, 2.72; N, 12.63. MS (FAB): *m/z* [M+1]^+^ 443.

*2-[2-((5-(4-Chloro-2-nitrophenyl)furan-2-yl)methylene)hydrazinyl]-4-(4-chlorophenyl)thiazole* (**11**): IR ν_max_ (cm^−1^): 3120.82, 3078.39 (aromatic C-H stretching), 2947.23, 2818.00 (C-H stretching), 1622.13, 1562.34, 1519.91, 1463.97 (C=N, C=C stretching and N-H bending), 1354.03 (C-H bending), 1257.59, 1222.87, 1116.78, 1093.64, 1010.70 (C-N, C-O stretching and aromatic C-H in plane bending), 923.90, 877.61, 825.53, 785.03, 734.88 (aromatic C-H out of plane bending). ^1^H-NMR δ (ppm): 6.97 (d, *J* = 3.6 Hz, 1H), 7.11 (d, *J* = 3.2 Hz, 1H), 7.43–7.49 (m, 3H), 7.83–7.89 (m, 3H), 7.93–7.95 (m, 2H), 8.13–8.14 (m, 1H), 12.37 (brs, 1H). Anal. Calcd. for C_20_H_12_Cl_2_N_4_O_3_S: C, 52.30; H, 2.63; N, 12.20; Found: C, 52.32; H, 2.61; N, 12.19. MS (FAB): *m/z* [M+1]^+^ 460.

*4-(4-Bromophenyl)-2-[2-((5-(4-chloro-2-nitrophenyl)furan-2-yl)methylene)hydrazinyl]thiazole* (**12**): IR ν_max_ (cm^−1^): 3383.14 (N-H stretching), 3116.97, 3078.39 (aromatic C-H stretching), 2943.37, 2856.58 (C-H stretching), 1620.21, 1558.48, 1516.05, 1467.83 (C=N, C=C stretching and N-H bending), 1357.89 (C-H bending), 1257.59, 1205.51, 1138.00, 1114.86, 1026.13, 1004.91 (C-N, C-O stretching and aromatic C-H in plane bending), 871.82, 821.68, 783.10, 734.88 (aromatic C-H out of plane bending). ^1^H-NMR δ (ppm): 6.98 (d, *J* = 3.6 Hz, 1H), 7.11 (d, *J* = 3.2 Hz, 1H), 7.45 (s, 1H), 7.62 (d, *J* = 8.4 Hz, 2H), 7.81–7.84 (m, 3H), 7.94 (d, *J* = 8.4 Hz, 1H), 7.98 (s, 1H), 8.12–8.13 (m, 1H), 12.37 (brs, 1H). Anal. Calcd. for C_20_H_12_BrClN_4_O_3_S: C, 47.68; H, 2.40; N, 11.12; Found: C, 47.67; H, 2.42; N, 11.10. MS (FAB): *m/z* [M+1]^+^ 504.

*2-[2-((5-(4-Chloro-2-nitrophenyl)furan-2-yl)methylene)hydrazinyl]-4-(p-tolyl)thiazole* (**13**): IR ν_max_ (cm^−1^): 3300.20 (N-H stretching), 3126.61, 3064.89 (aromatic C-H stretching), 2918.30, 2858.51 (C-H stretching), 1622.13, 1562.34, 1525.69, 1465.90 (C=N, C=C stretching and N-H bending), 1352.10 (C-H bending), 1257.59, 1222.87, 1138.00, 1114.86, 1028.06 (C-N, C-O stretching and aromatic C-H in plane bending), 923.90, 881.47, 813.96, 785.03, 765.74, 731.02 (aromatic C-H out of plane bending). ^1^H-NMR δ (ppm): 2.33 (s, 3H), 6.96–7.11 (m, 2H), 7.18–7.28 (m, 3H), 7.73–8.14 (m, 6H), 12.34 (brs, 1H). Anal. Calcd. for C_21_H_15_ClN_4_O_3_S: C, 57.47; H, 3.44; N, 12.77; Found: C, 57.46; H, 3.44; N, 12.78. MS (FAB): *m/z* [M+1]^+^ 439.

*2-[2-((5-(4-Chloro-2-nitrophenyl)furan-2-yl)methylene)hydrazinyl]-4-(4-methoxyphenyl)thiazole* (**14**): IR ν_max_ (cm^−1^): 3203.76 (N-H stretching), 3140.11, 3082.25 (aromatic C-H stretching), 2937.59, 2833.43 (C-H stretching), 1625.99, 1560.41, 1523.76, 1508.33, 1462.04 (C=N, C=C stretching and N-H bending), 1359.82, 1346.31 (C-H bending), 1249.87, 1184.29, 1116.78, 1026.13 (C-N, C-O stretching and aromatic C-H in plane bending), 829.39, 813.96, 742.59 (aromatic C-H out of plane bending). ^1^H-NMR δ (ppm): 3.79 (s, 3H), 6.96–6.99 (m, 3H), 7.11 (d, *J* = 3.6 Hz, 1H), 7.19 (s, 1H), 7.77–7.95 (m, 5H), 8.13–8.14 (m, 1H), 12.35 (brs, 1H). Anal. Calcd. for C_21_H_15_ClN_4_O_4_S: C, 55.45; H, 3.32; N, 12.32; Found: C, 55.45; H, 3.33; N, 12.35. MS (FAB): *m/z* [M+1]^+^ 455.

### 3.3. Bioassays

#### 3.3.1. Anticandidal Activity

Anticandidal activity assay was performed against* Candida albicans* (ATCC 10231), *Candida utilis* (NRRLY 900), *Candida tropicalis* (NRRLY 12968), *Candida krusei* (NRRLY 7179), *Candida parapsilosis* (NRRLY 12696) and *Candida glabrata* (ATCC 2001) standard strains according to the CSLI method [37].

The minimum inhibitory concentration (MIC, µg/mL) values against pathogenic *Candida* strains were determined by a broth microdilution method using a 96-well plate format [37,38]. Compounds **1**–**14** and fluconazole as a standard control were first dissolved in dimethyl sulfoxide (DMSO) (25%) at an initial concentration of 2000 µg/mL.

All *Candida* strains were inoculated on Potato Dextrose Agar (PDA) prior the experiments at 37 °C. After the incubation, sufficiently grown microorganisms were inoculated in sterile saline (0.85%), and then standardized according to the turbidity to 5 × 10^3^ CFU (McFarland No: 0.5) per well in RPMI medium under sterile conditions. Serial dilutions were prepared in 100 µL RPMI medium with an equal amount of the test samples, and 100 µL each microorganism suspension was pipetted into each well and incubated at 37 °C for 24 h. Positive growth controls (to assess the presence of turbidity) were performed in wells without the test samples, whereas the negative growth control (medium) was also evaluated. MIC was deﬁned as the lowest concentration without any visible growth of the yeast when compared with the growth in the control plate. All experiments were performed in duplicate.

#### 3.3.2. Cytotoxicity

The tetrazolium salt, MTT (3-(4,5-dimethylthiazol-2-yl)-2,5-diphenyltetrazolium bromide), is used to measure the metabolic activity of viable cells. Tetrazolium salts are reduced to formazan by mitochondrial succinate dehydrogenase, an enzyme which is only active in cells with an intact metabolism. The formazan can be quantified photometrically and it is in correlation with the number of viable cells [39]. Cytotoxicity was tested using the MCF-7 human breast adenocarcinoma cell line and NIH/3T3 mouse embryonic fibroblast cell line. NIH/3T3 and MCF-7 cells were incubated in RPMI medium (Hyclone, Thermo Scientific, Logan, UT, USA) supplemented with fetal calf serum (Hyclone), 100 IU/mL penicillin and 100 mg/mL streptomycin (Hyclone) at 37 °C in a humidified atmosphere of 95% air and 5% CO_2_. MCF-7 and NIH/3T3 cells were seeded at 10,000 cells into each well of 96-well plates. After 24 h of incubating period, the culture mediums were removed and compounds were added to culture medium at 3.9–500 µg/mL concentrations. After 24 h of incubation, cytotoxicity tests were performed using the MTT assay, which measures mitochondrial activity, in MCF-7 and NIH/3T3 cells. Firstly, 20 µL MTT solution (5 mg/mL MTT powder in PBS) was added to each well. After 3 h incubation period at 37 °C, 5% CO_2_, the contents of the wells were removed and 100 µL DMSO was added to each well. Then, OD of the plate was read at 570 nm. Inhibition% was calculated for each concentration of the compounds. IC_50_ values were estimated by non-linear regression analysis. Cisplatin was used as a positive control. Stock solutions of compounds were prepared in DMSO and further dilutions were made with fresh culture medium. All experiments were performed in duplicate.

## 4. Conclusions

In the present paper, we have described the synthesis of some new thiazolyl hydrazone derivatives, which were investigated for their anticandidal effects and cytotoxicity against the MCF-7 and NIH/3T3 cell lines. Due to their low toxicity toward NIH/3T3 cells (IC_50_ > 500 µg/mL), compound **14** can be considered as the most effective antifungal derivative against *C. utilis* with a MIC value of 250 µg/mL when compared with fluconazole (MIC = 2 µg/mL), whereas compound **11** can be identified as the most promising anticancer agent against the MCF-7 cancer cell line with an IC_50_ value of 125 µg/mL, compared with cisplatin (IC_50_ = 31.2 µg/mL). In view of these results, further research should be carried out on the development of new effective anticancer agents by suitable modification of compound **11**.

## Supplementary Materials

Supplementary materials can be accessed at: http://www.mdpi.com/1420-3049/19/9/14809/s1.

## Supplementary Files

Supplementary File 1
