# Supplementary material for: Synthesis and in Vitro Evaluation of New Nitro-Substituted Thiazolyl Hydrazone Derivatives as Anticandidal and Anticancer Agents
Source: Molecules. 2014 Sep 17;19(9):14809–20. doi: 10.3390/molecules190914809 (PMC6271239; doi:10.3390/molecules190914809)

Supplementary Materials

Figure S1. IR Spectrum of compound **1**.

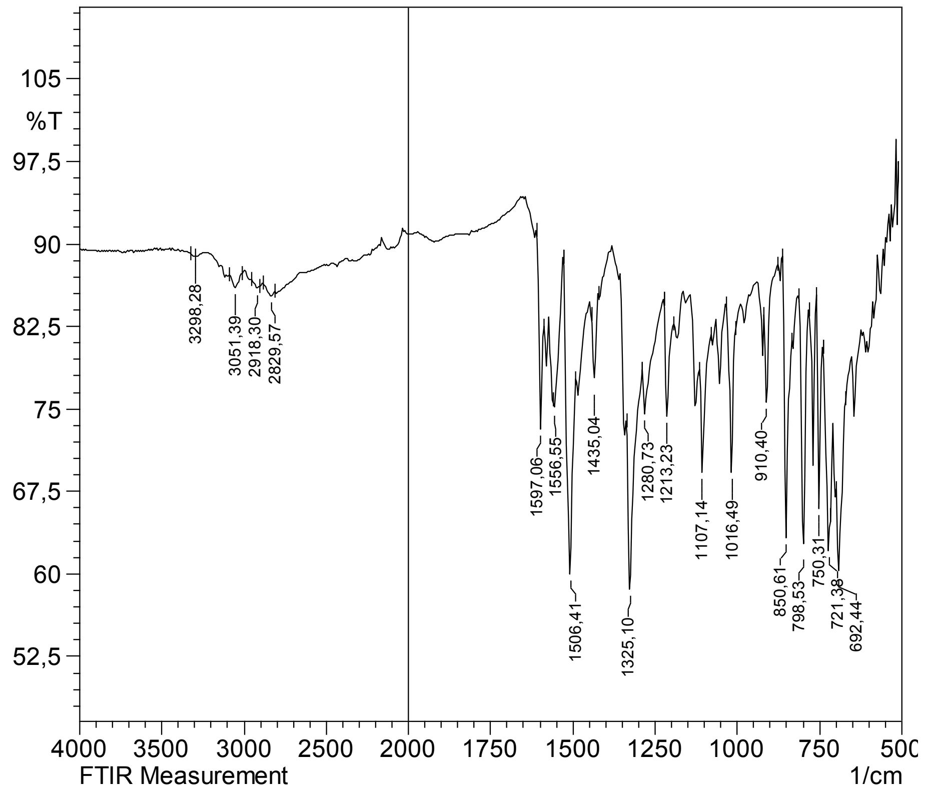

Figure S2. IR Spectrum of compound **2**.

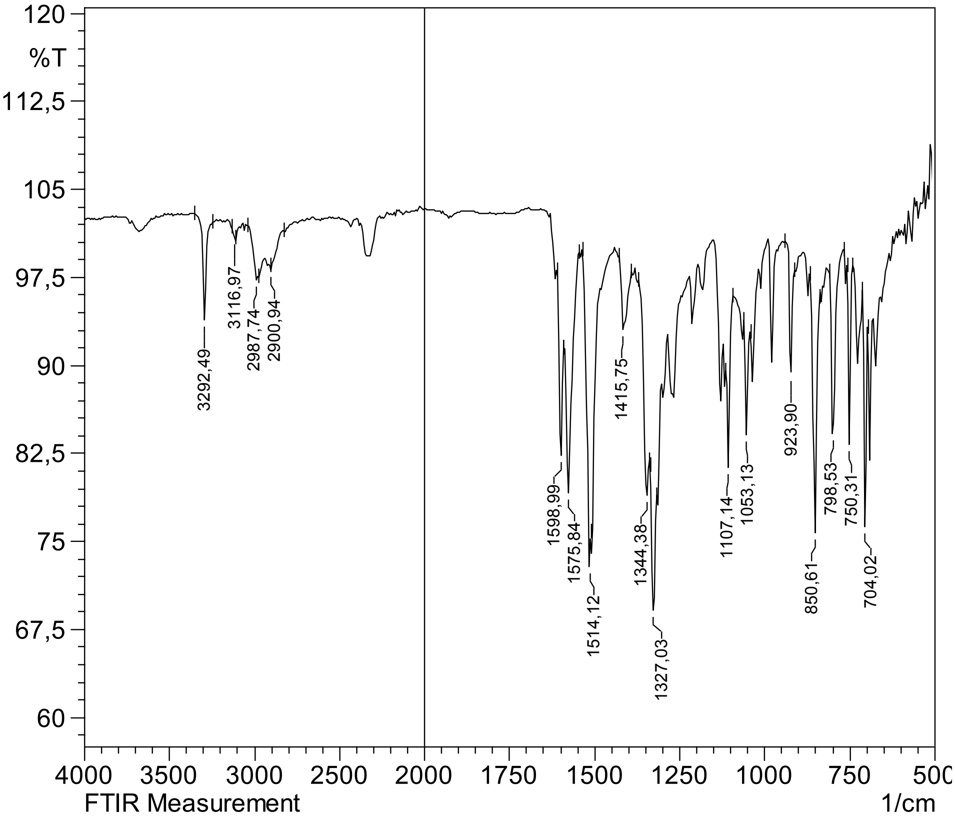

Figure S3. IR Spectrum of compound **3**.

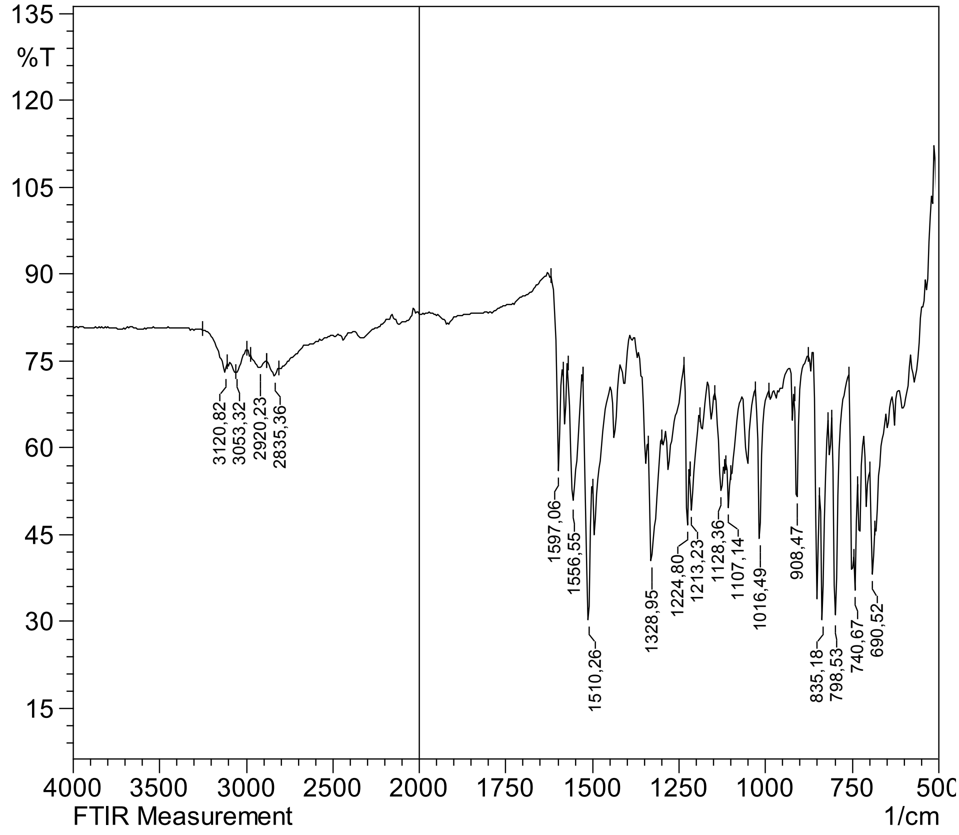

Figure S4. IR Spectrum of compound **4**.

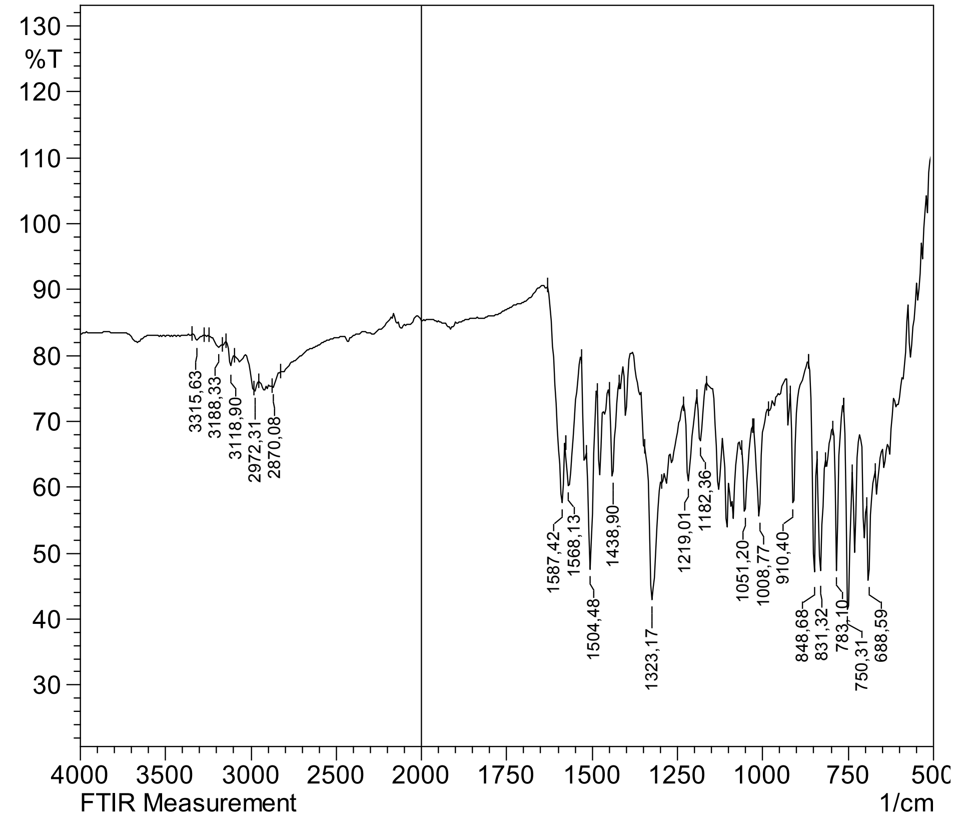

Figure S5. IR Spectrum of compound **5**.

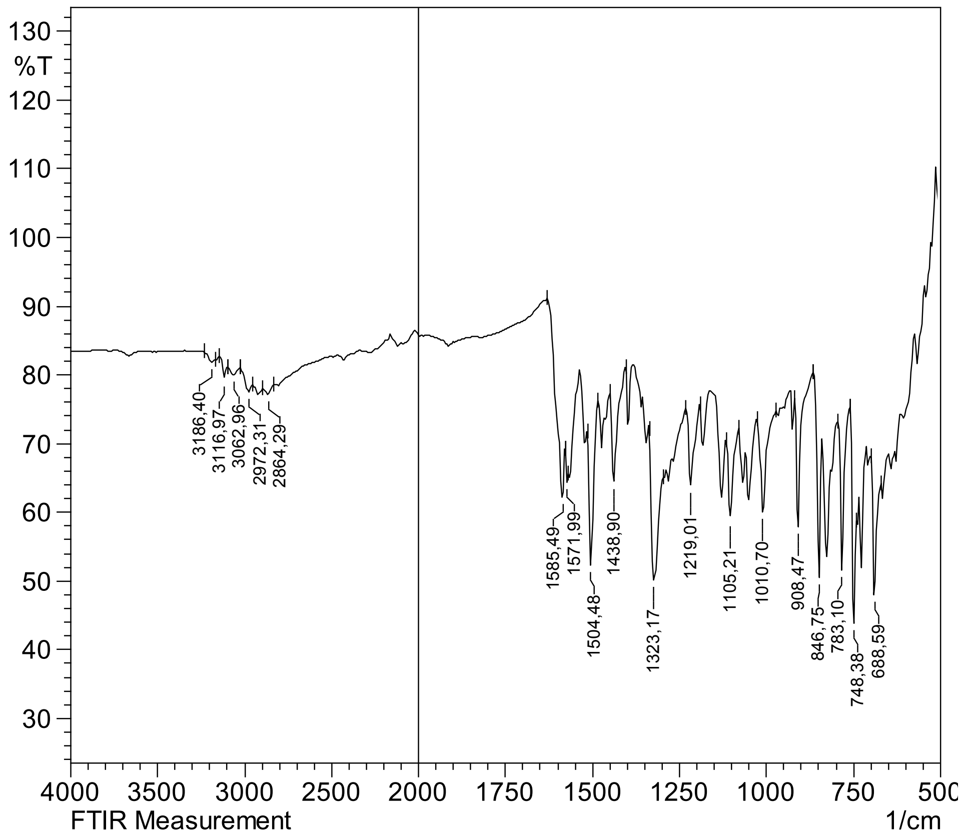

Figure S6. IR Spectrum of compound **6**.

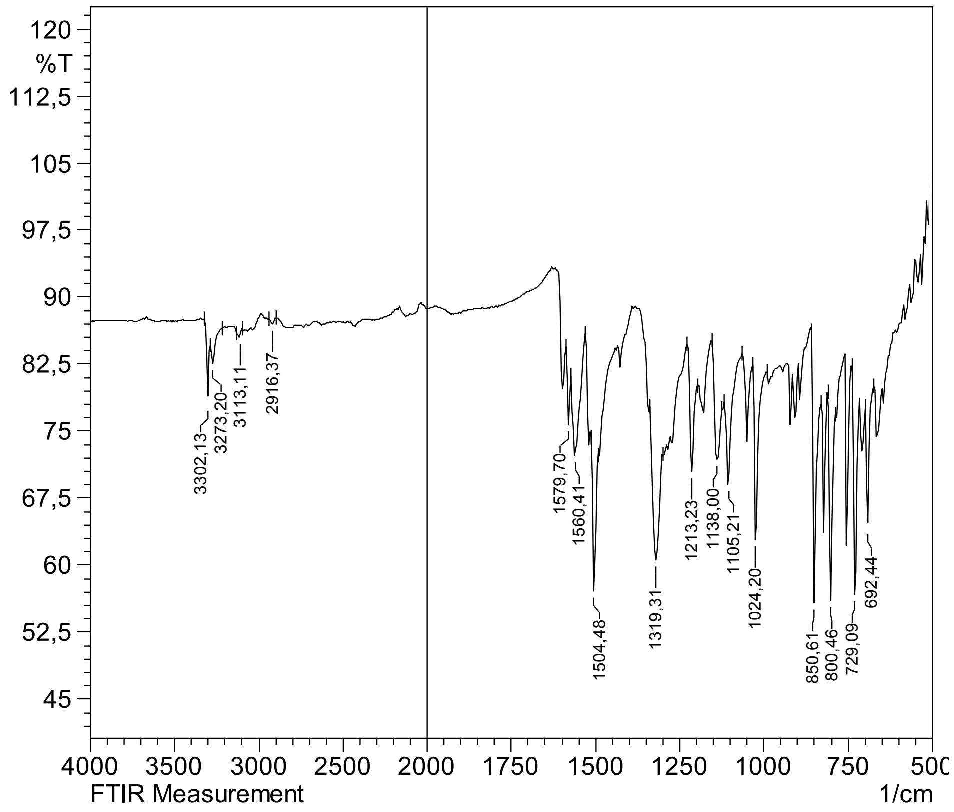

Figure S7. IR Spectrum of compound **7**.

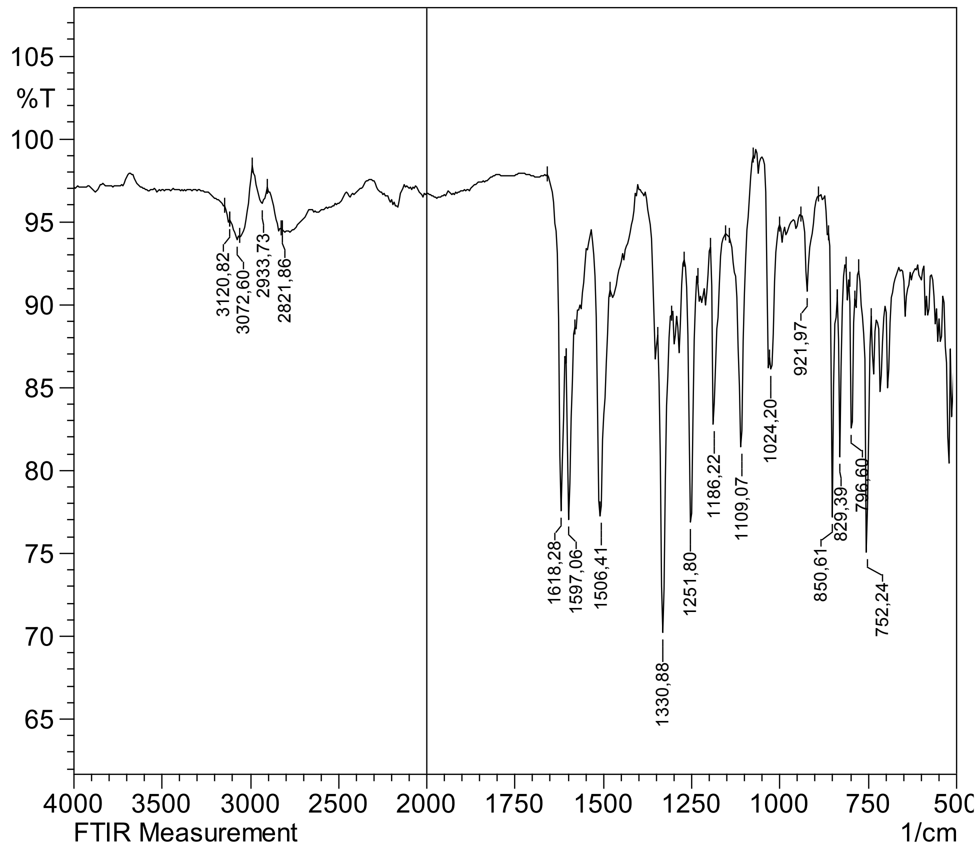

Figure S8. IR Spectrum of compound **8**.

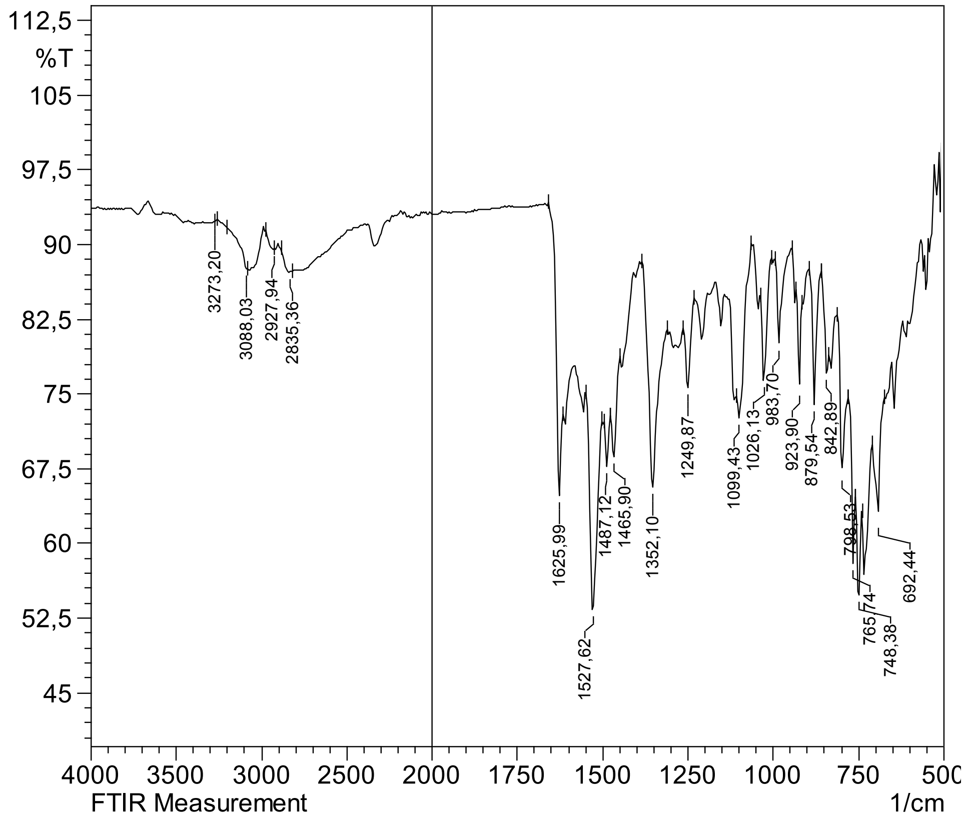

Figure S9. IR Spectrum of compound **9**.

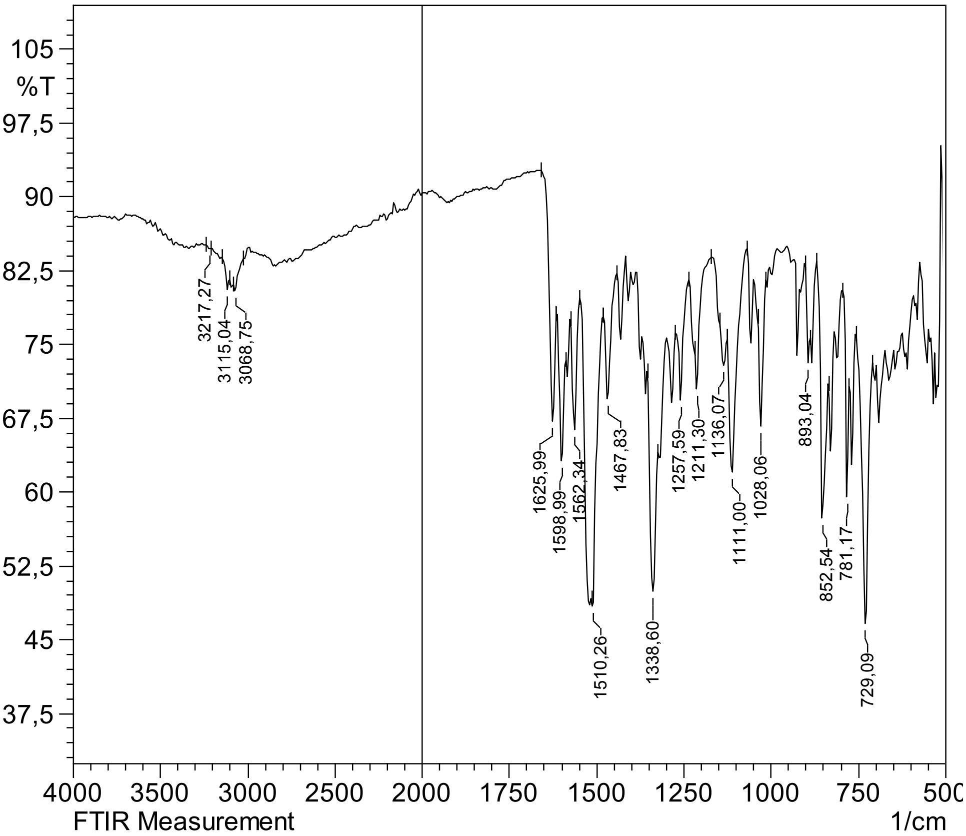

Figure S10. IR Spectrum of compound **10**.

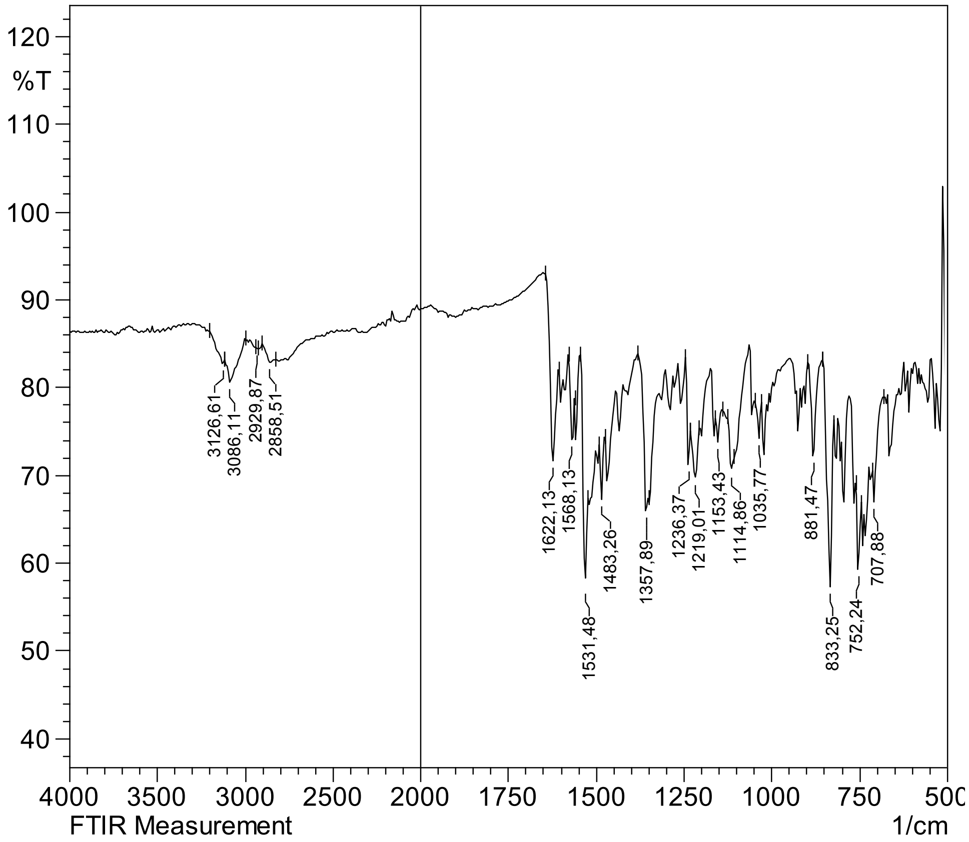

Figure S11. IR Spectrum of compound **11**.

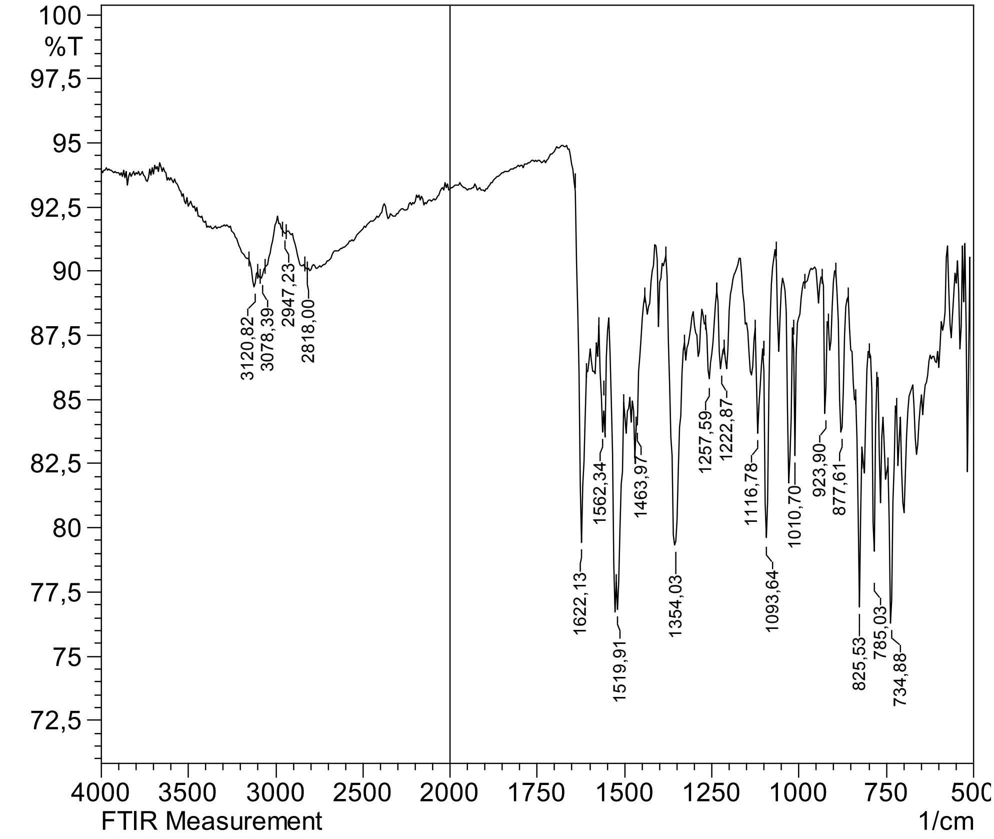

Figure S12. IR Spectrum of compound **12**.

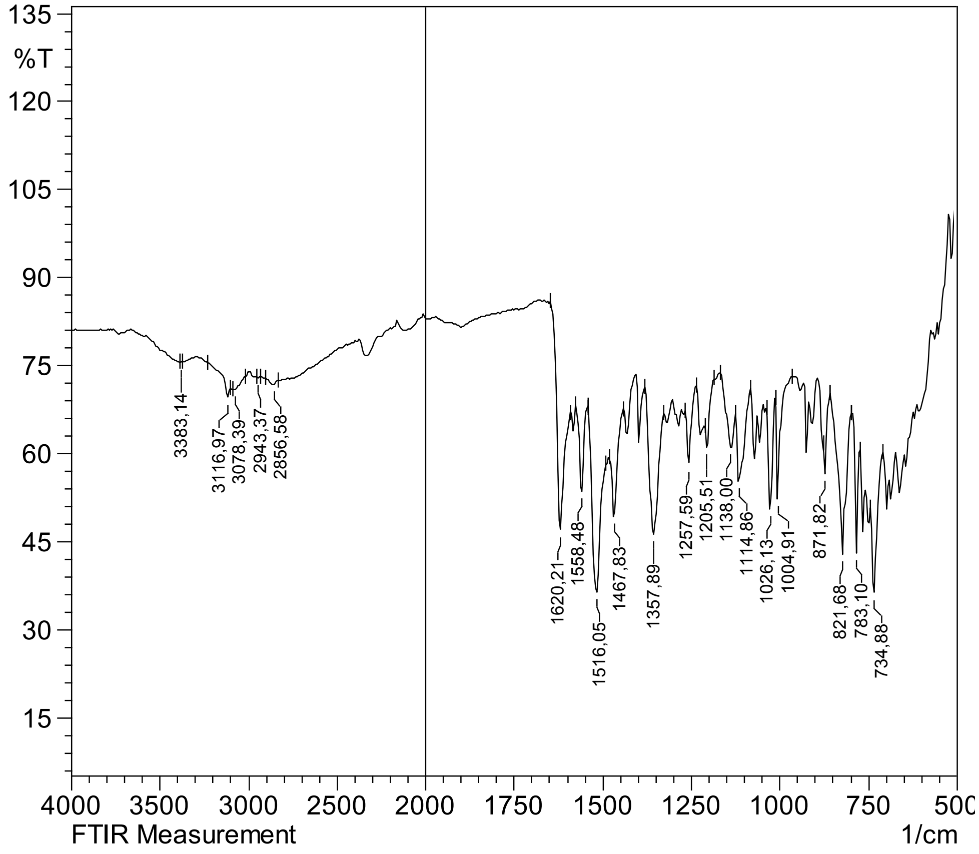

Figure S13. IR Spectrum of compound **13**.

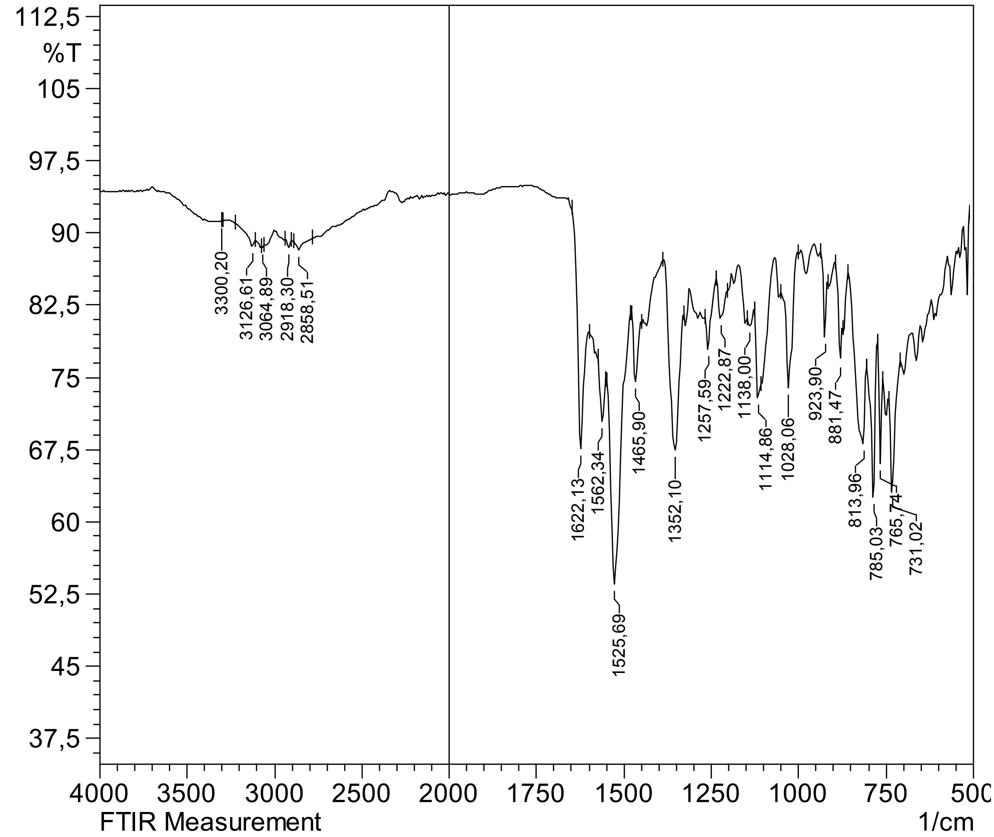

Figure S14. IR Spectrum of compound **14**.

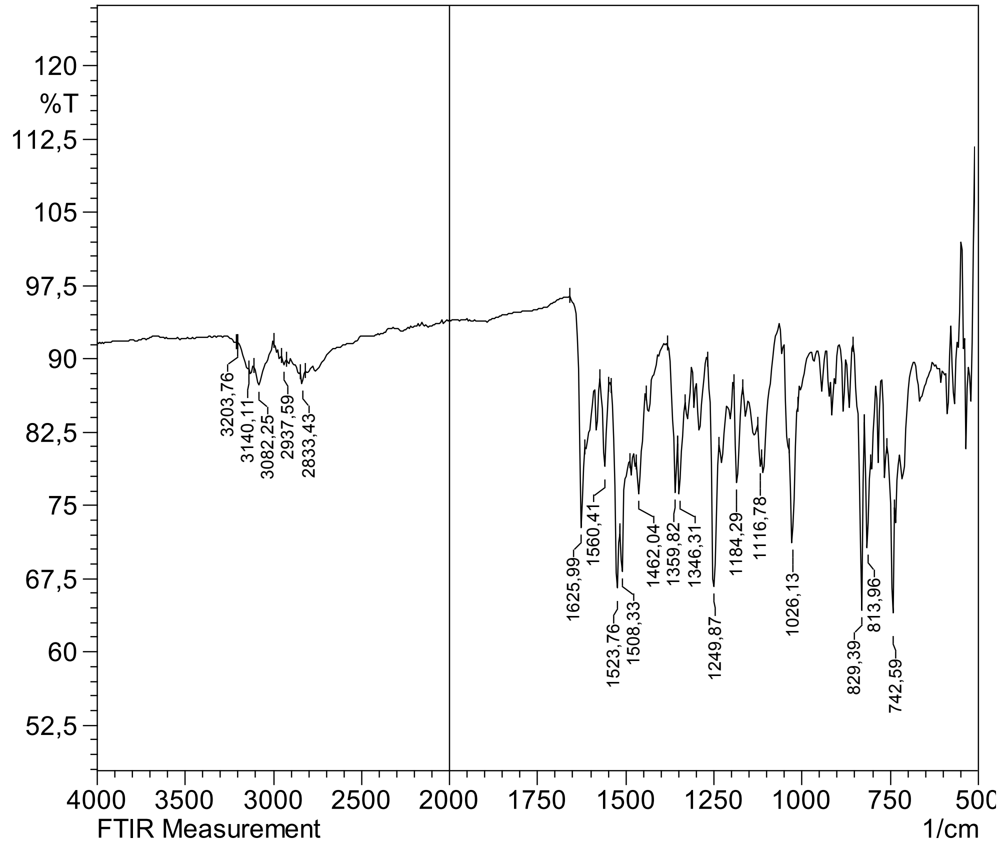

Figure S15. ^{1}H-NMR spectrum of compound **1**.

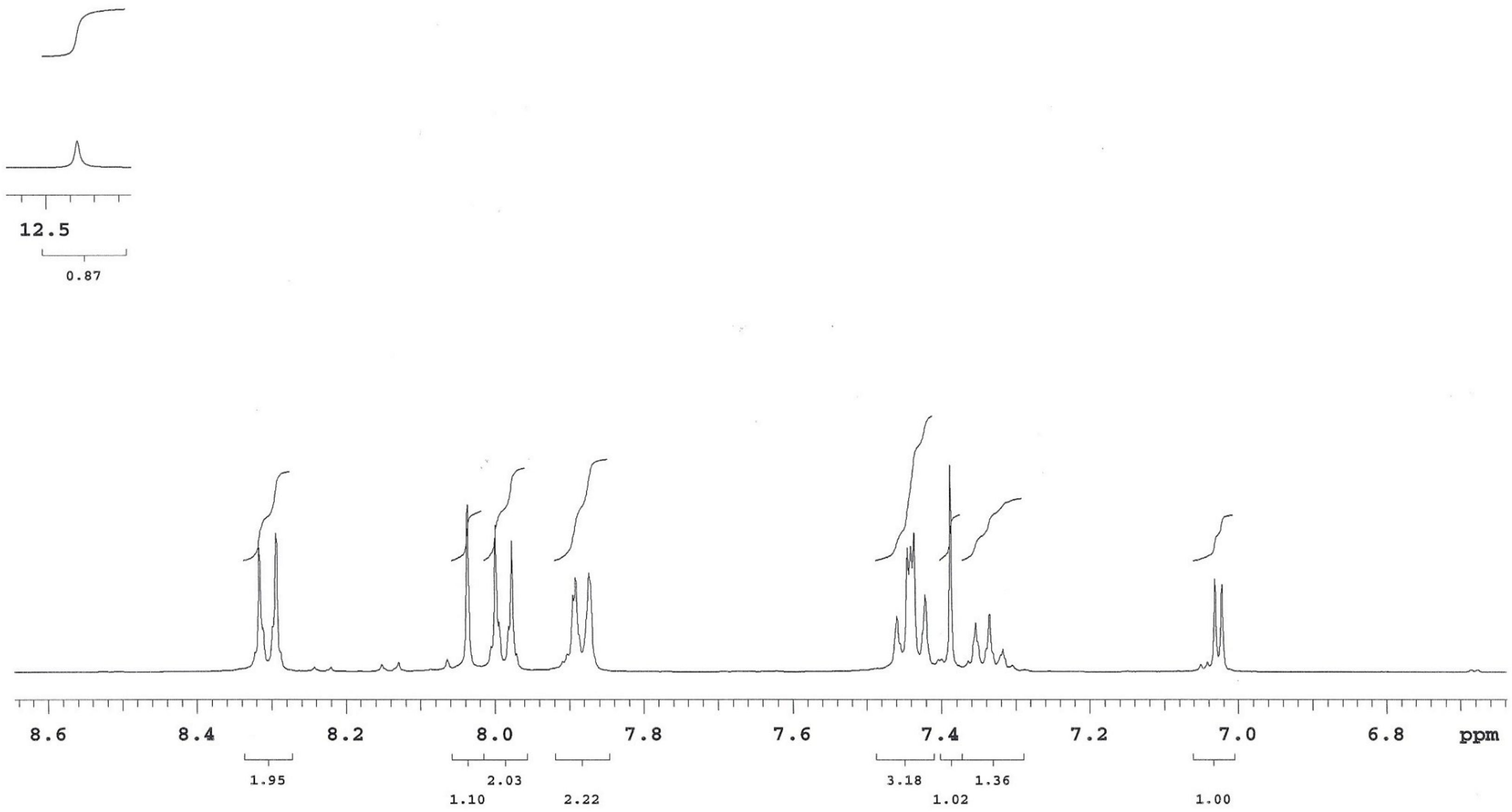

Figure S16. ^{1}H-NMR spectrum of compound **2**.

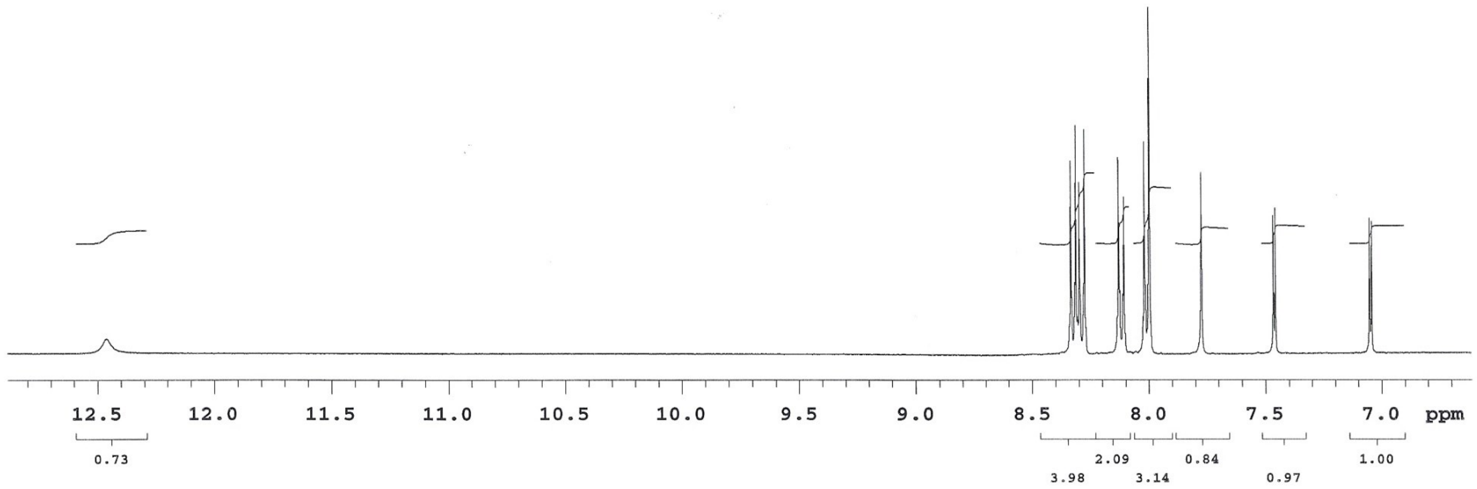

Figure S17. ^{1}H-NMR spectrum of compound **3**.

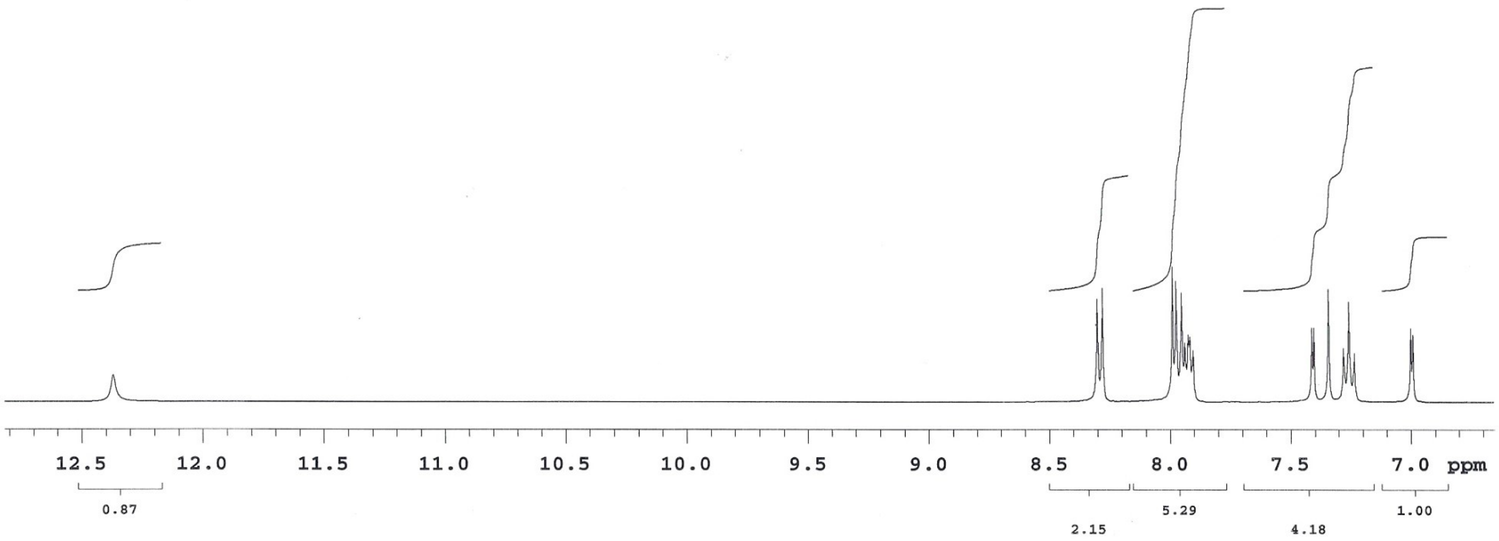

Figure S18. ^{1}H-NMR spectrum of compound **4**.

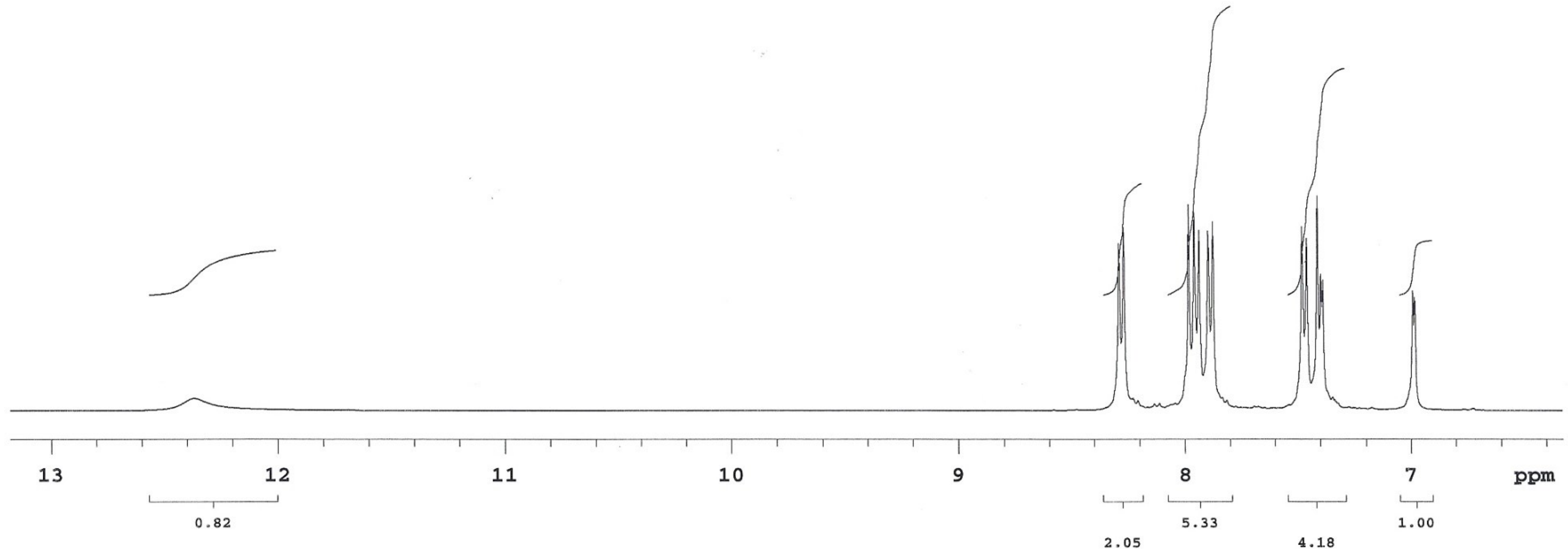

Figure S19. ^{1}H-NMR spectrum of compound **5**.

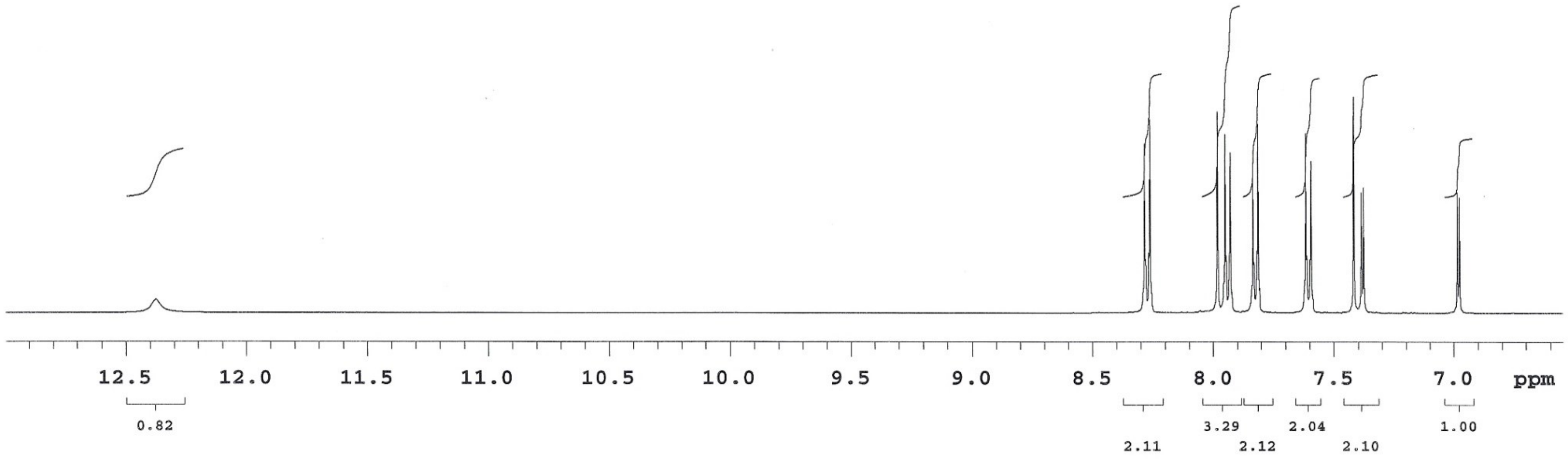

Figure S20. ^{1}H-NMR spectrum of compound **6**.

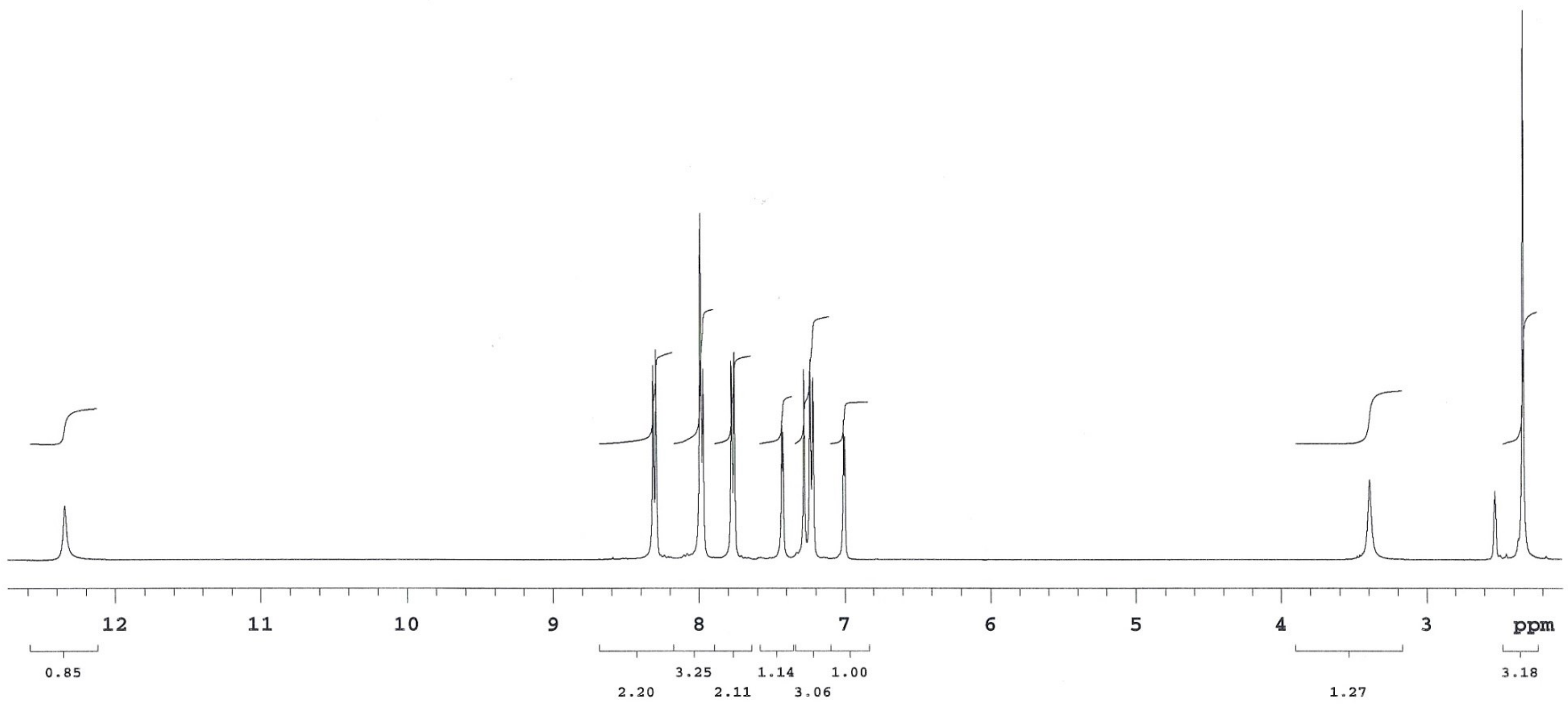

Figure S21. ^{1}H-NMR spectrum of compound **7**.

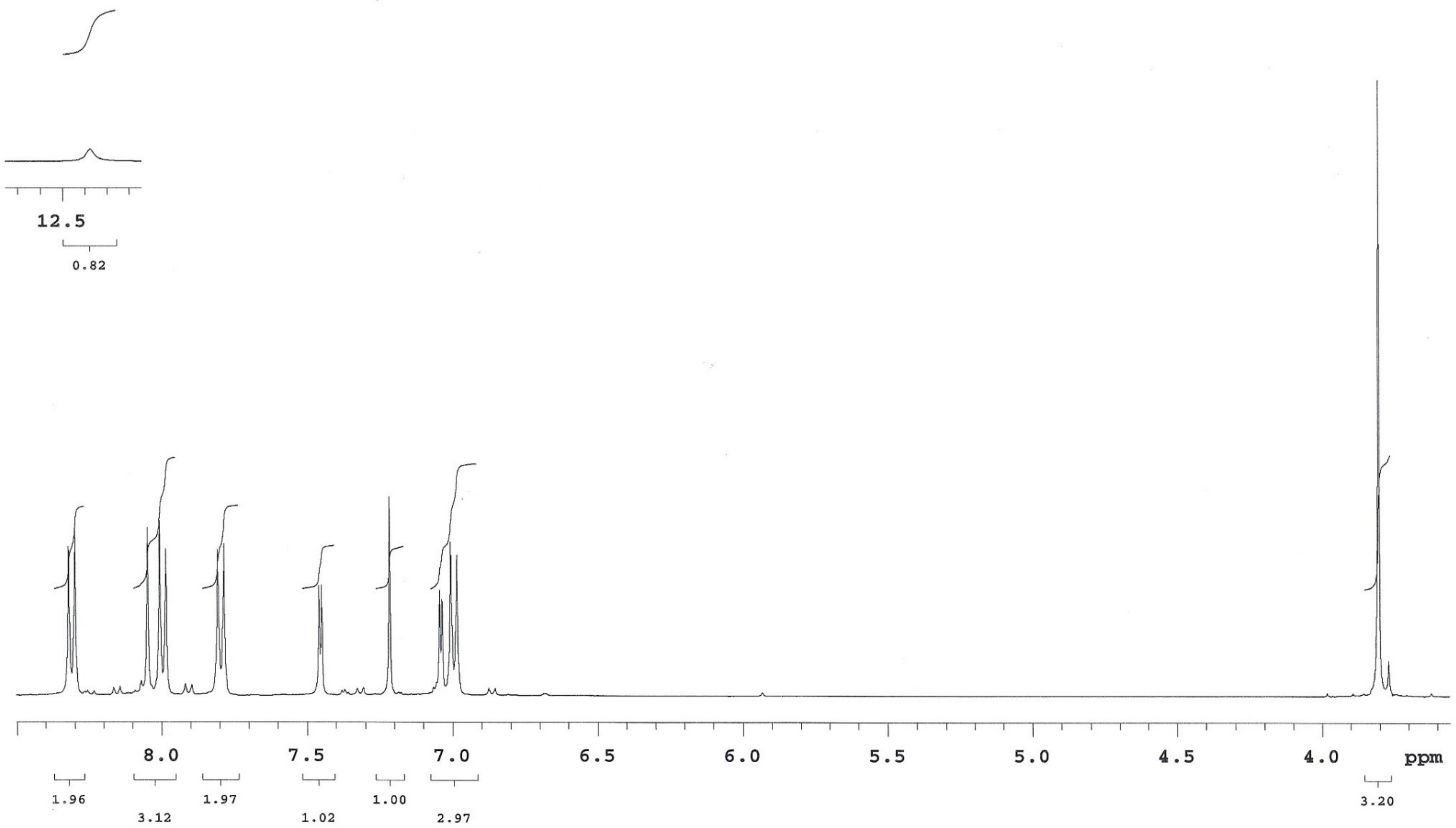

Figure S22. ^{1}H-NMR spectrum of compound **8**.

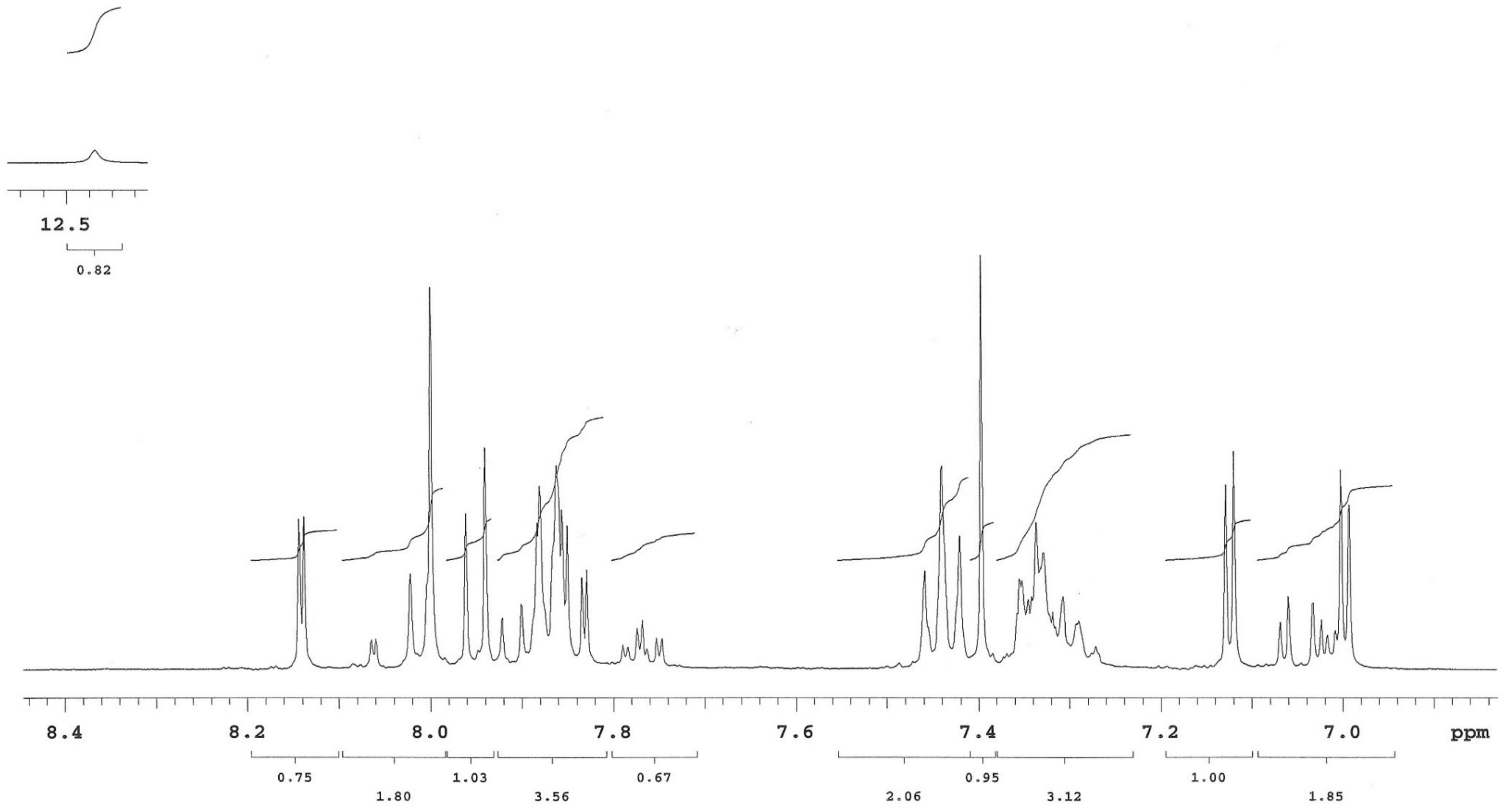

Figure S23. ^{1}H-NMR spectrum of compound **9**.

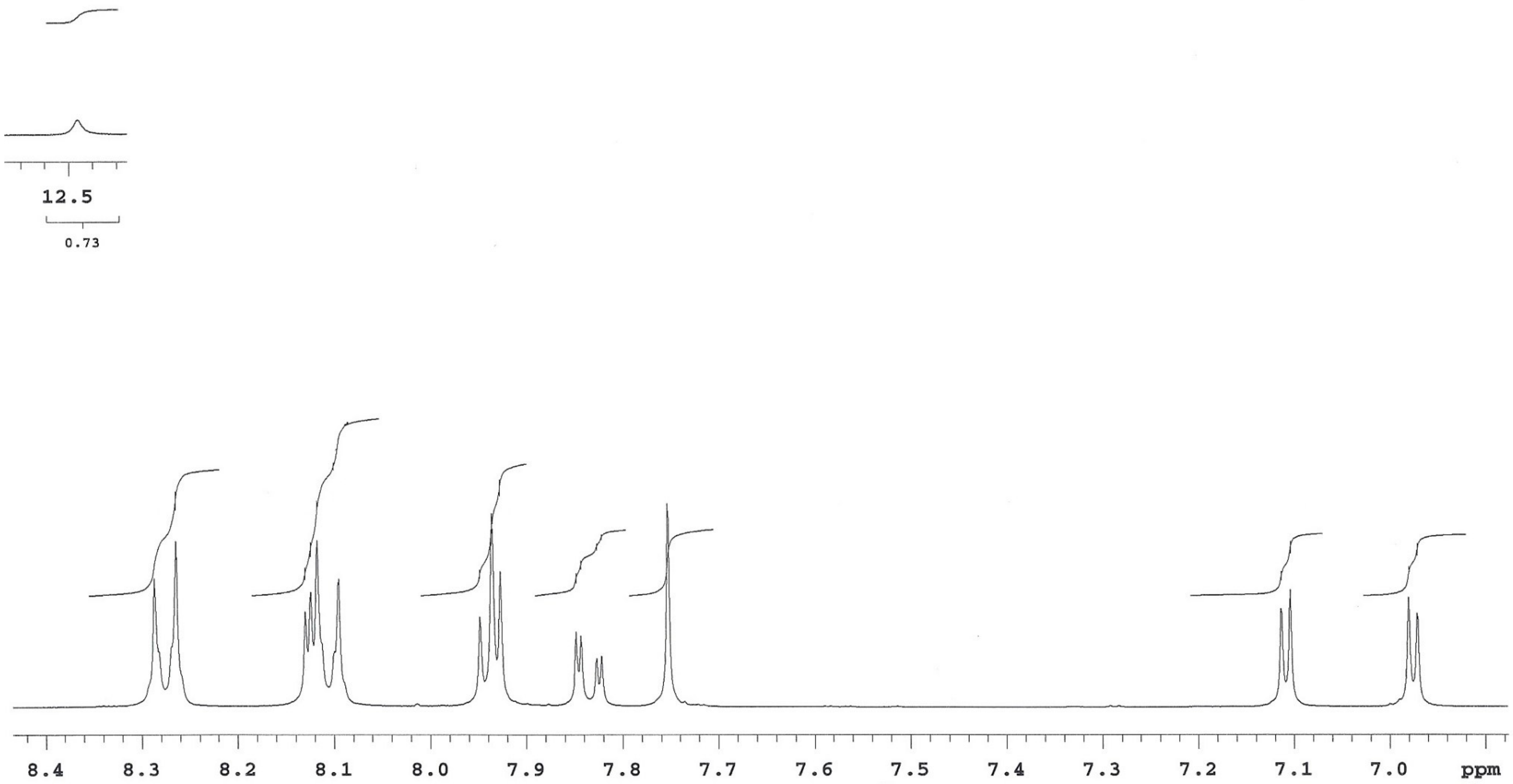

Figure S24. ^{1}H-NMR spectrum of compound **10**.

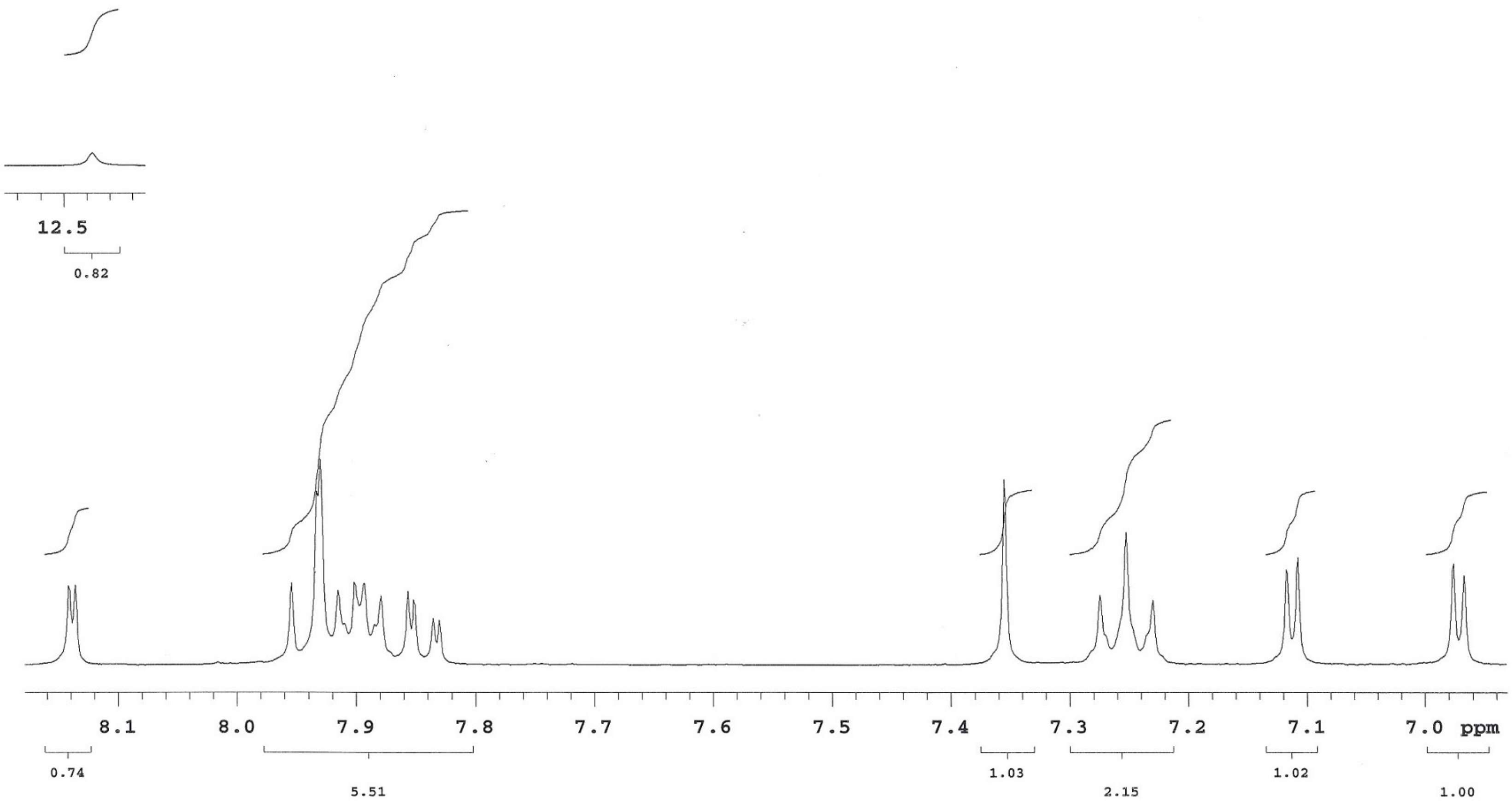

Figure S25. ^{1}H-NMR spectrum of compound **11**.

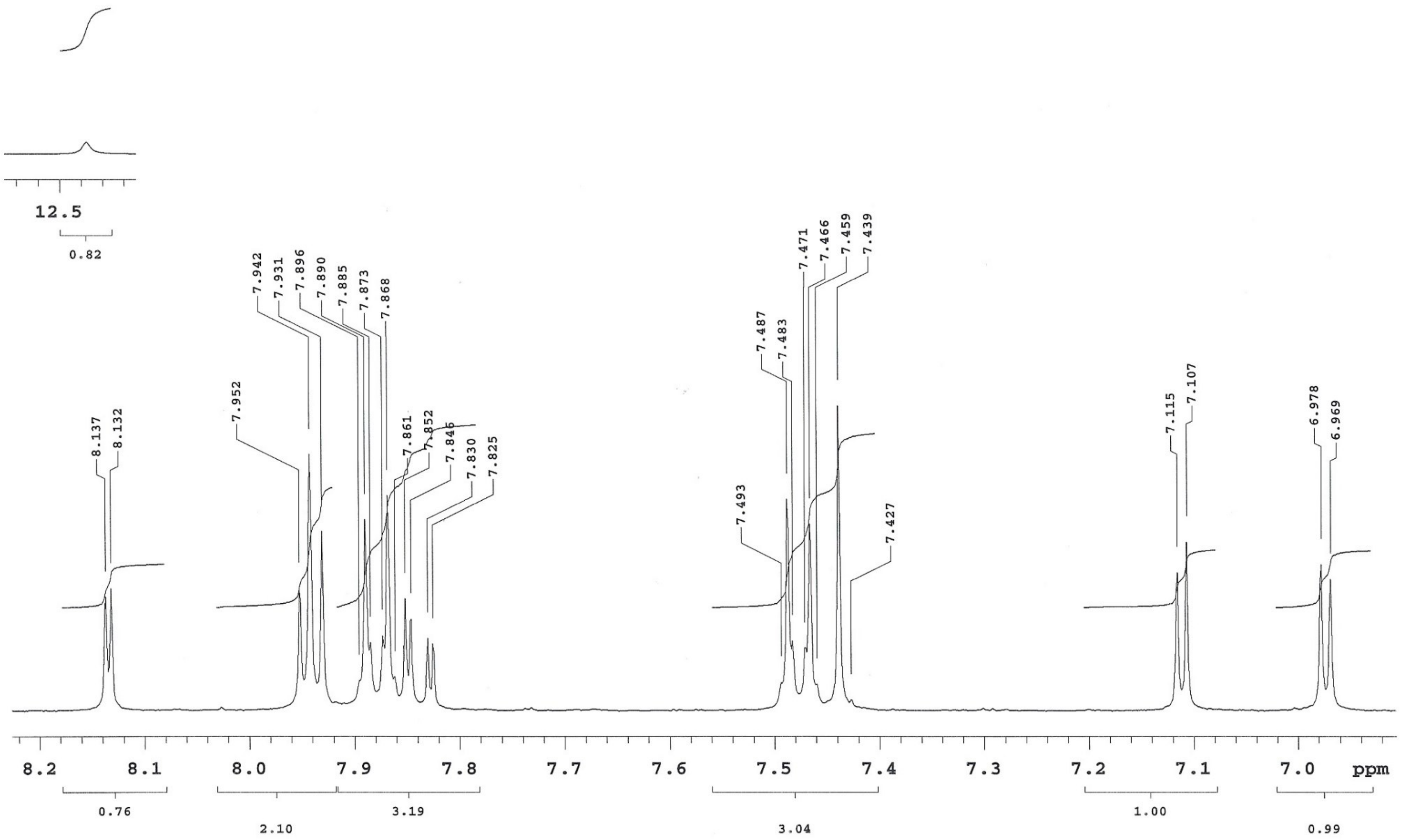

Figure S26. ^{1}H-NMR spectrum of compound **12**.

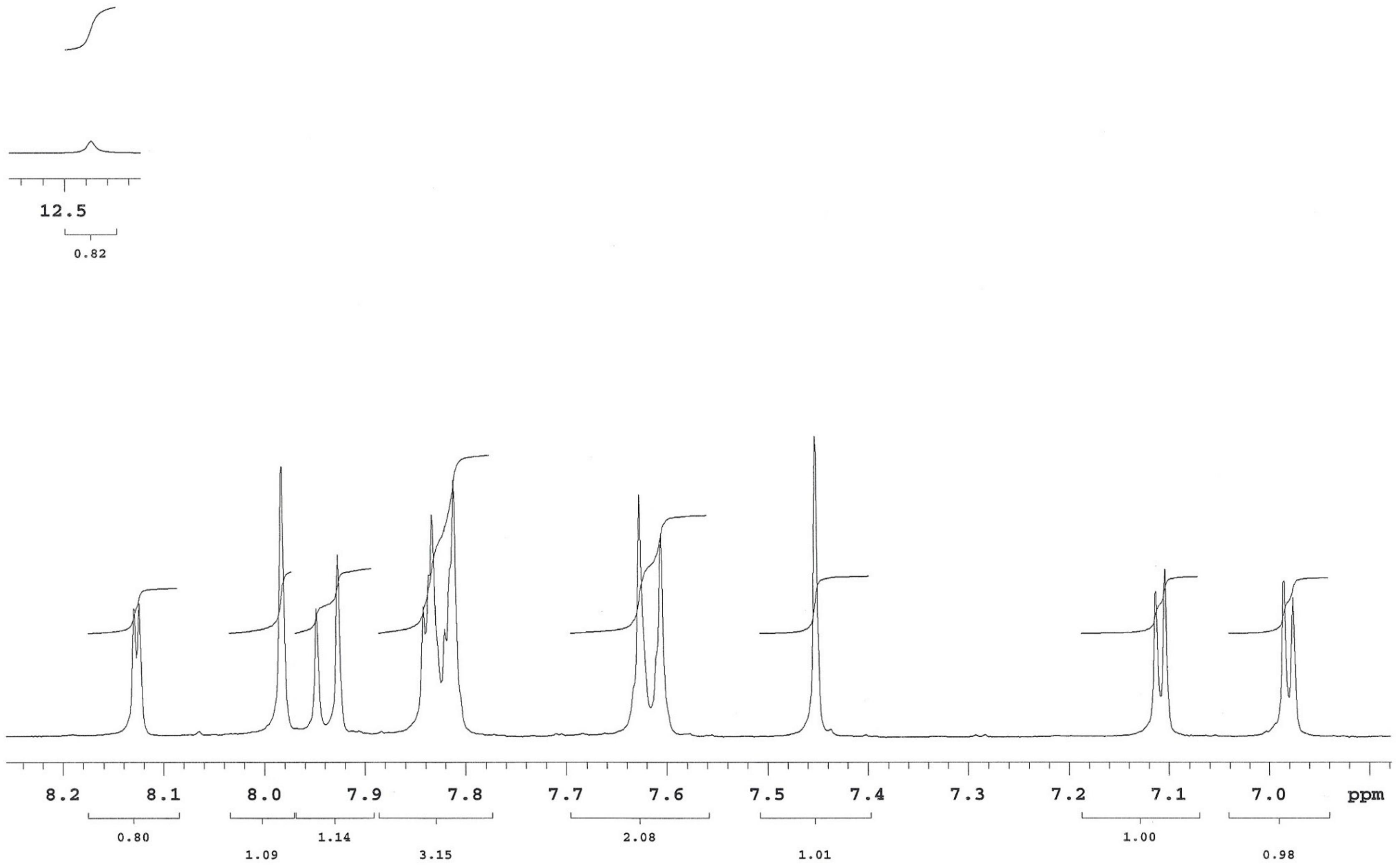

Figure S27. ^{1}H-NMR spectrum of compound **13**.

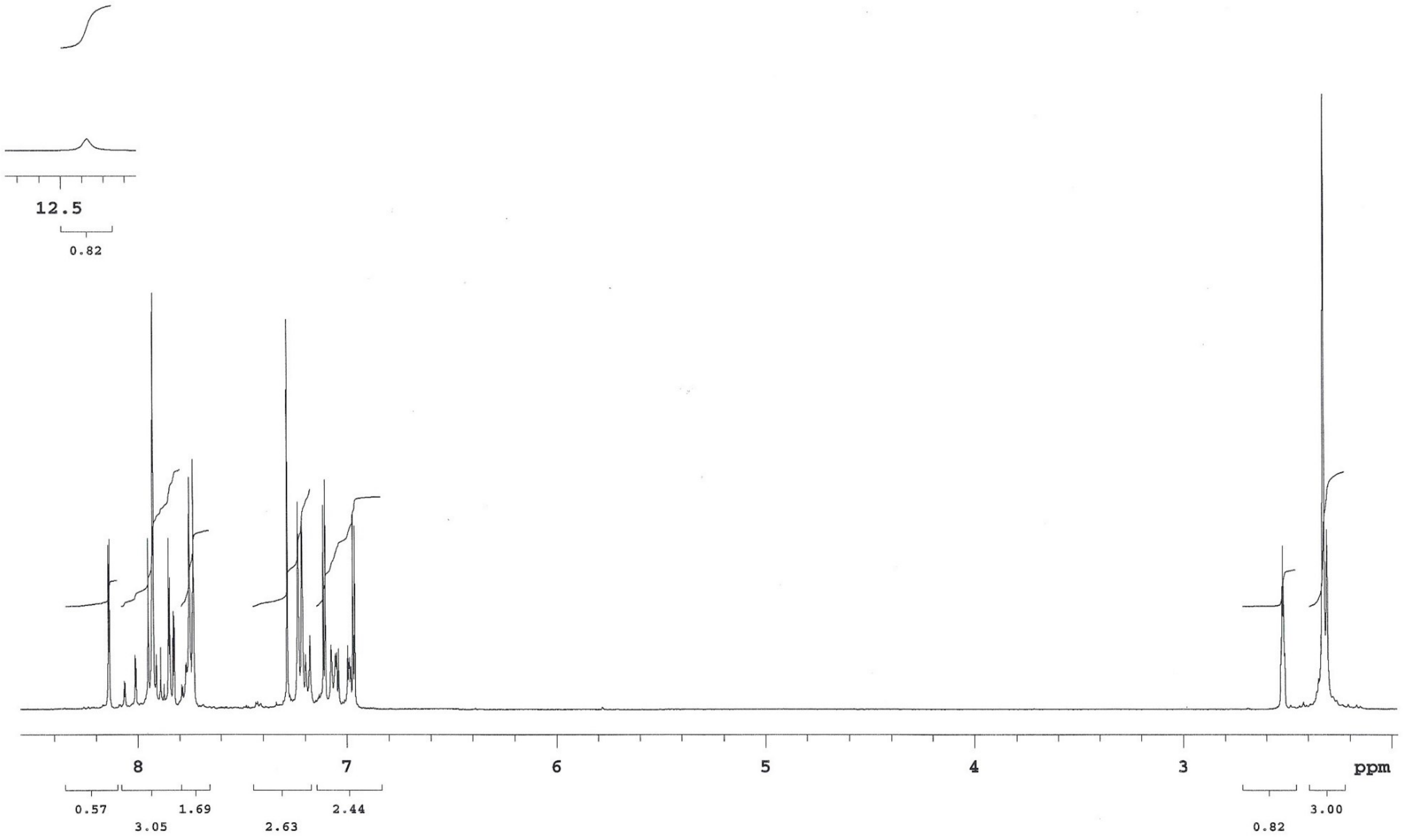

Figure S28. ^{1}H-NMR spectrum of compound **14**.

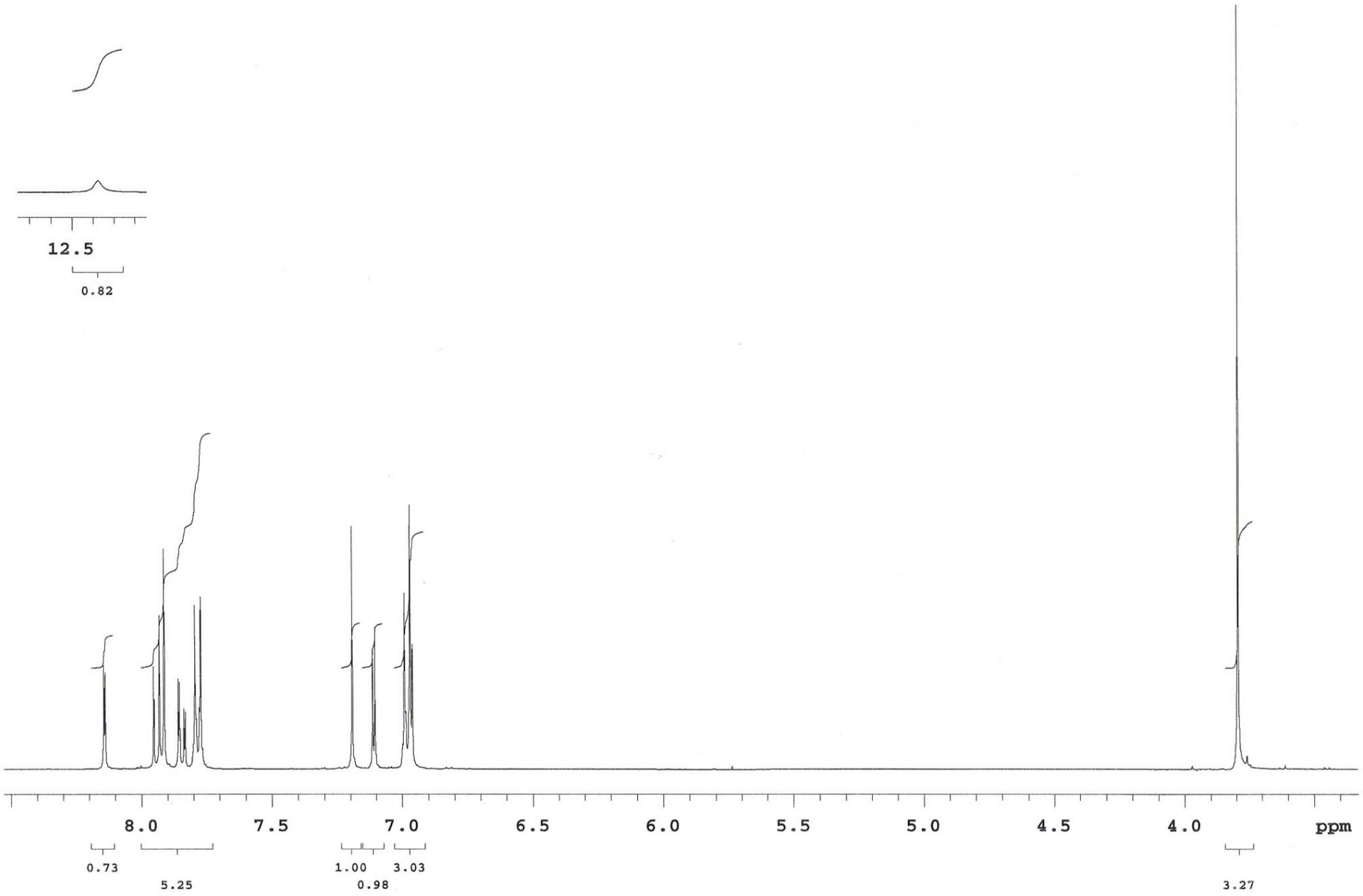

Supplement: Supplementary File 1 [file molecules-19-14809-s001.pdf]
